# Design and testing of the key technology of the cotton direct seeding machine

**DOI:** 10.3389/fpls.2025.1530725

**Published:** 2025-02-20

**Authors:** WEI Song, Liu Hongyun

**Affiliations:** ^1^ College of Engineering, Huazhong Agricultural University, Wuhan, China; ^2^ Department of Architectural Engineering, Guizhou Industry Polytechnic College, Guiyang, China

**Keywords:** cotton, sowing depth regulation, design, experiments, mechanical design

## Abstract

The paper aims to slove the problems of low sowing depth qualification index and poor seeding stability during in summer direct-sown cotton in the Yangtze River Basin China. To this end, a cotton seeder with rotary tillage and electric drive seeding was designed. In order to satisfy the agronomic requirements of summer direct-sown cotton, the key structural parameters of this planter were determined, and the key factors affecting the stability of seeding depth were derived. The test method of rotary orthogonal was adopted, with the initial traction angle, initial spring increment, and Working speed of the machine serving as the test factors, and the sowing depth qualification index and sowing depth consistency coefficient of variation serving as the test indexes. The results demonstrated when the initial traction angle was set to 0°. the initial spring increment was 15mm, and the Working speed was 3km·h^−1^, the sowing depth qualification index was obtained to be 95.59%, and the sowing depth consistency coefficient of variation was 7.38%. A test was conducted to assess the accuracy of the speed control of the electric drive seed dispenser of the sowing system. The plant spacing was set at 15, 18, and 20 cm, and the speed was set at 4.01-6.10 km·h^−1^. The results demonstrated the overall mean adjustment time for the speed of the seed dispenser disk was 1.04 seconds, with a standard deviation of 0.36; the overall mean steady-state error was 0.28 r/min, with a standard deviation of 0.21 r/min; the overall mean control accuracy was 98.86%, with a standard deviation of 1.01. A field test was conducted with a set plant spacing of 15 cm and a seeder speed that was not fixed. The sowing plant spacing was able to focus on the set plant spacing within the range of the qualified grain spacing (15 ± 3 cm) of the emergence of seedlings. The average sowing emergence Conformity index was 86.48%, with a minimum of 83.81%.

## 1 Introduction

Cotton is a material with a wide range of applications, including in the textile, industrial, medical, and other sectors. The cotton market and economy are inextricably linked to the stability of the agricultural and textile industries (De Siqueira et al., 2024; Yemadje et al., 2025; Zhao et al., 2023). The quality of the cotton sowing has a major impact on yield, as cotton seeds are less capable of breaking the soil than wheat and corn. The soil in the cotton-growing regions of the Yangtze River basin is characterized by high moisture content and low permeability, which can impede the effective operation of seeders. The presence of straw stubble in the sowing plots further complicates the process, as it increases the number of openings in the soil surface that the seeder must traverse to create the seed trench. The resulting furrow walls are often uneven, which can cause the seeds to fall into the trench and bounce or roll significantly as they descend. Furthermore, the traditional passive wheel-driven seed dispenser is susceptible to slippage, which can result in leakage and other undesirable outcomes. This, in turn, can impact the consistency of seed sowing depth and the uniformity of grain spacing and row spacing (Chen et al., 2004; Fallahi and Rapufat, 2008; Ahmad et al., 2015).

Precision sowing technology ensures that seeds are deposited into the soil at a predetermined location in accordance with the specified spacing (plant spacing), row spacing, and sowing depth, thereby satisfying the agronomic requirements. This process guarantees a consistent sowing depth and uniform spacing (plant spacing).The evaluation of the quality of sowing is dependent upon two important indicators: the consistency of sowing depth and spacing, and row spacing uniformity. In recent years, numerous experts and scholars from both domestic and international contexts have conducted comprehensive studies on the impact of consistency in sowing depth and spacing, as well as row spacing uniformity, on crop yield (Karayel and Zmerzi, 2008; Canakc et al., 2009; Ozmerzi et al., 2002; Busari et al., 2015). The formation of the sowing depth is the result of the combined action of the furrow opener, soil return, and mulch compaction. Of these factors, the consistency of the furrow opener depth is the most important influencing factor. Vamerali et al. (2006) designed a wide-winged sharp-angle no-tillage furrowing imitation furrow opener, which solved the problem of insufficient soil compactness in the seed bed. In recent years, many experts and scholars have conducted in-depth studies on the effects of sowing depth consistency as well as grain and row spacing uniformity on crops. Liu et al., 2024 developed a downforce measurement and control system based on a “T”-shaped furrow opener. The impact of soil dynamic parameters and downforce from the furrow opener on furrowing depth was examined, and a test platform was constructed to validate the precision of the system in measuring downforce and regulating furrowing depth. Cao et al. (2022) employed gas-solid coupling simulation technology to study the influence of the structural parameters of the air spring on the downforce of the furrowing monobloc. Optimization of the structural parameters of the air spring was conducted, resulting in enhanced consistency and uniformity of the depth of furrowing. The term “uniformity” is used to describe a quality or state of being consistent and without variation. Mahapatra et al., 2024a developed an electronically operated cotton planter, which Quality sowing seed index and precision index values of 88.8 and 12.75%. He et al. (2023) sought to address the issue of poor-quality wheat furrowing in the Huanghuaihai region by developing a device that imitates the seed furrow suppression trenching technique. This device is designed to ensure uniform suppression after sowing. Jia et al. (2018) designed a synchronized furrowing suppression device for use in the northeastern region, where sticky soil and poor furrowing quality are prevalent. This device can be opened over the smooth “V”-shaped seed furrow, creating a high-quality seed furrow environment for optimal seed germination. Boulakia et al. (2018) designed a slide knife type integrated open furrow opener, which facilitates the flow of seeds into the bottom of the furrow before the soil. This addresses the issue of the traditional opener disturbing the soil flow into the seed furrow, which has been identified as a challenge in ensuring consistent sowing depth. Jinqing et al. (2020) designed a potato furrow opener sowing depth regulation device that employs a distinct profiling furrow configuration. This device addresses the shortcomings of traditional potato furrowing, including inadequate adaptability and suboptimal overall profiling, and is tailored to meet the specific requirements of diverse regional contexts. In addition, companies such as Deere, Precision Planting, and Kinze have introduced furrowers with an air spring and a lower pressure control system. This has been done in order to improve the quality of furrowing under no-tillage conditions, to enable the monitoring and adjustment of lower pressure in single rows, to improve the stability of high-speed furrowing, and to increase the yield of maize (John, 2022; Precision Planting, 2022; Kinze, 2022).

The existing cotton direct seeding machine is generally unsuitable for use in wheat cotton rotation areas, where there is a large amount of rainfall and heavy soil, and where viscous conditions prevail. In such circumstances, it is difficult to ensure the requisite quality of furrowing. In light of these considerations, tongs this paper proposes the development of an integrated cotton direct seeding machine that incorporates a rotary plow stubble precision furrowing mechanism. At the same time, in order to reduce the number of implements to work in the field, the preparation of the ground and sowing operations are integrated into one compound operation. The rotary plow stubble device is designed to facilitate the crushing and loosening of the previous crop straw, thereby creating an open border furrow and enhancing the structural integrity of the seedbed. Furthermore, the incorporation of a seeding depth control device enables the attainment of consistent seeding depths. A control system for precise cotton seeder, based on”radar speed measurement + real-time motor control,” was designed., When the system was working, the MCU analyzed the data from each sensor and generated motor control commands to drive DC motor to drive the rotation of the seed metering shaft, so as to realize the matching of seed metering shaft speed and tractor forward speed, and to realize the stepless seeding amount adjustment. The system met the requirements of cotton precision seeding and can provide a reference for the improvement of the structure of speed⁃dependent control system of precision cotton planters.

## 2 Structure and working principle of the whole machine

### 2.1 Machine structure and furrowing agronomic requirements

#### 2.1.1 Agronomic requirements

The cotton planting mode in the Yangtze River Basin region eliminates the need for seedling transplantation, reduces the application of fertilizer, increases the planting density of cotton, and has numerous advantages. These include simplified field management, reduced planting costs, and a reduction in environmental pollution. The planting mode offers comprehensive economic benefits. This approach effectively alleviates the contradiction between grain and cotton for land, facilitates the recovery of the Yangtze River Basin planted cotton area, stabilizes cotton production in the Yangtze River Basin, and plays an important role in promoting the high-quality development of the cotton industry. It is of significant importance.

Wheat is harvested in mid to late May to take advantage of moisture-sowing opportunities. The use of a seeding machine allows for the completion of the entire compartment grooving in a single operation. Seed bed rotary tillage, sowing, mulching, and other processes are also conducted. The use of basal fertilizer, pest control, and weed management strategies is avoided to prevent complications. Pre-seedling spraying herbicides are applied to control weeds. The one-time harvest of cotton occurs in early November, as illustrated in **Supplementary Figure 1**.

**Figure 1:**
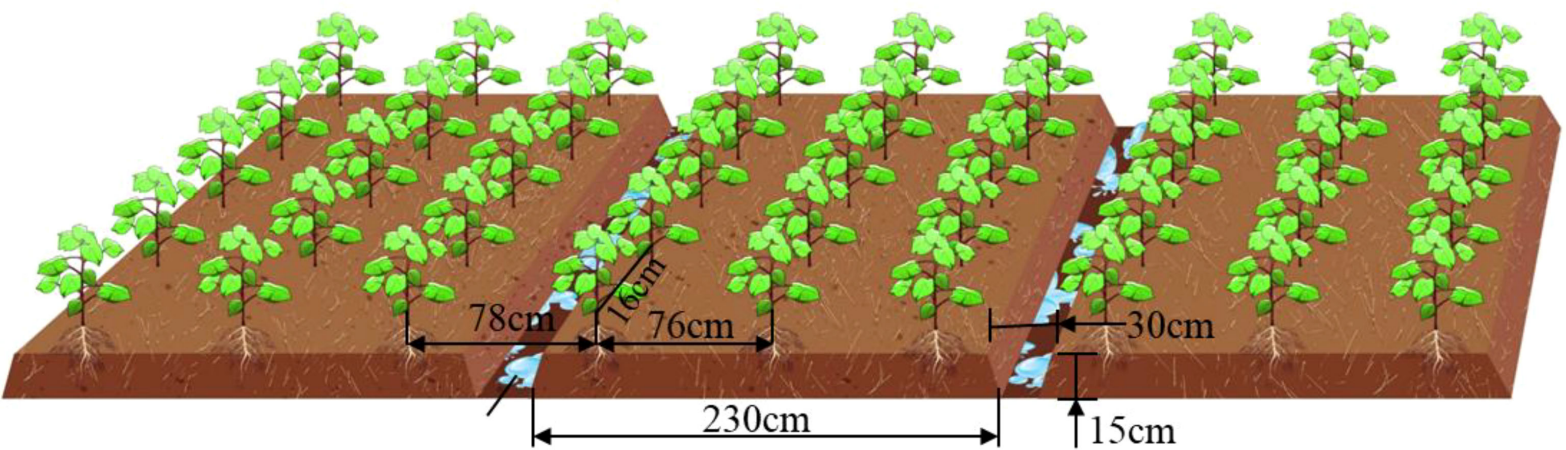
Effect of operation in the cotton area of the Yangtze River Basin.

The impact of cotton seeding operations in the Yangtze River Basin region is illustrated in **Figure 1**. The figure depicts three rows of a compartment with a row spacing of 76 cm, equal rows of planting, a compartment width of 230 cm, a compartment surface on both sides of the open bed ditch to facilitate drainage, a bed ditch depth of 15 cm, a width of 30 cm, The distance between the adjustment of each hole is 15cm, with one seed of direct-seeded cotton treated with finishing coatings sown in each. The depth of sowing is 3 cm.

#### 2.1.2 Structure of the whole machine

**Figure 2:**
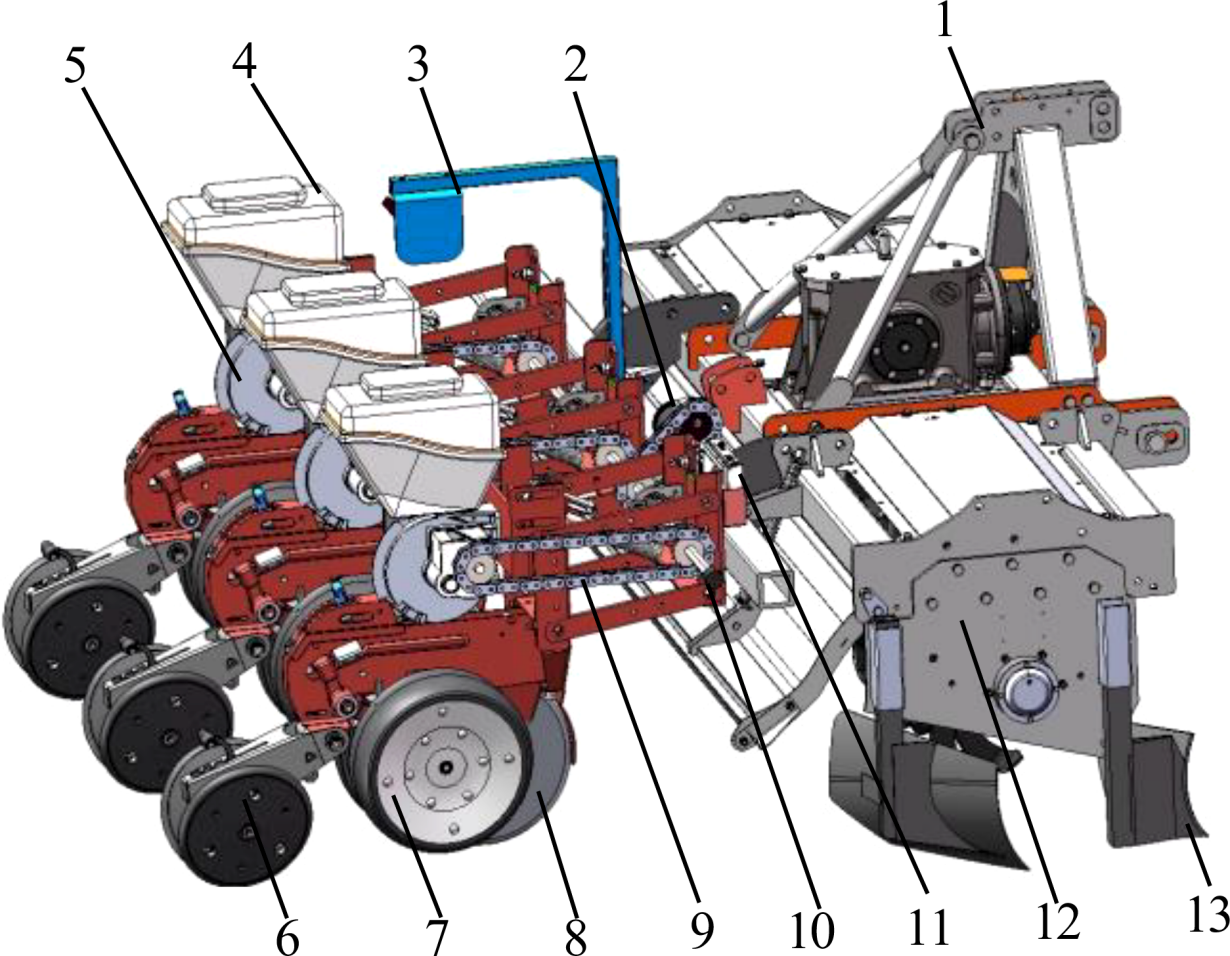
Structural diagram of seeder. 1. Three-point suspension device 2. Brushed DC motor 3. Radar speed measuring device 4. Seed box 5. Seed dispenser 6. “V” type suppression mulching device 7. Depth limiting wheel 8. Double disc opener 9. Transmission chain 10. Speed measuring encoder 11. Main controller 12. Rotary ploughing device 13. Sharp angle furrow opener imitating the shape of boots.

The configuration of the cotton planter is illustrated in **Figure 2**. It primarily comprises a three-point hitch, a transmission apparatus, a frame, a furrowing unit, a rotary plow, a transmission component, and other essential elements. The rotary tillage unit is installed on both sides of the frame, while the profiling frame is installed at the rear of the frame using the four-link profiling method. The cotton seeding unit is located at the rear of the profiling frame and consists of a furrow opener, seed box, seed discharger, suppression wheel, and other components. The furrow opener and seed discharger utilize an electric drive mode for transmission. The machine is capable of performing a comprehensive range of operations, including seedbed cleaning, furrowing, mulching, and suppression, in a single cycle. The key technical specifications are presented in **Table 1**.

**Table 1:** Main technical parameters of seeder.

Parameters	Value
Dimension (*L×W×H*)/(mm×mm×mm)	2100×2500×850
Number of lines of work/line	3
Sowing row spacing/mm	760
Ditching depth/mm	2~4
Working width/mm	2300
Working speed/(km·h^−1^)	3~8
Matching power/kW	≥90

### 2.2 Working principle and main technical parameters

The planter is drawn forward by the tractor during operation. Double disc opener is responsible for opening the seed furrow, while the profiling spring, profiling adjusting screw, and profiling tie rod adjust the profile. The rotary plowing device is driven by the tractor power output shaft, with the purpose of finely breaking up the soil, loosening the soil, and swirling the stubble soil mixture. The stubble soil mixture is then incorporated into the tilled soil to establish an optimal seed bed environment. Concurrently, the front and rear combined furrow plows, positioned on both sides of the border furrow, facilitate drainage. Simultaneously, the cotton seed is dispensed from the pneumatic seed dispenser discharge port into the built-in seed guide tube, thereby completing the seed casting operation. The machine’s Working speed is measured using a radar system that operates in real time. Concurrently, the cotton seeds are discharged from the pneumatic seed discharger’s discharge port into the built-in seed guide tube of the double disc opener, thereby completing the seeding operation. The entire speed control system is powered by the tractor battery. The system includes a speed radar that acquires real-time data on the equipment’s Working speed. The seeding control terminal is configured according to the predefined seeding mu sowing volume and plant spacing. This configuration is integrated with the equipment’s Working speed and other relevant information. The terminal drives the seeding motor rotation, thereby regulating the speed of the seeding operations.

## 3 Key component design and parameters

### 3.1 Rotary tillage and stubble destroyer

Wheat straw stubble covers a considerable portion of the soil, rendering it adhesive and difficult to cultivate. The cotton furrowing machine is prone to encountering difficulties when operating in such soil conditions, particularly when attempting to clear obstructions. The rotary plow and furrow operation exhibits excellent quality, resulting in a smooth soil surface after sowing, uniform sowing depth, and effective resolution of the Yangtze River Basin paddy area’s challenging soil crushing and time constraints. The rotary plow and furrow compound operation represents a valuable approach to enhancing the field passability of the cotton furrowing machine. The positive rotary tiller is capable of disrupting the topsoil and creating a uniform soil flow that is then dispersed backward, thereby facilitating the systematic distribution of seed fertilizer (Cheng et al., 2021). The speed of stubble eradication achieved by the stubble-eradicating rotary cultivator is contingent upon a number of factors, including the Working speed of the furrowing machine tool, the radius of the rotary cultivator, the depth of the rotary cultivator, and the rotation speed of the rotary cutter shaft. Provided that the stubble extinguishing operation is satisfactory—that is, provided that a certain plowing depth and minimum stubble extinguishing speed are ensured—it can be observed that the larger the rotary cutter shaft rotation speed, the smaller the rotary plowing radius. The optimal design for the knife roller is a low speed and a large diameter, as much as possible (Cheng et al., 2023). In this paper, the minimum stubble knife speed is 4 m/s, the stubble height H is 100 mm, the rotary stubble radius is 300 mm, the minimum rotary plow rotation speed n is 245 r/min, and the rotary plow is operated in positive rotation. The open-bed furrow device employs a boot-shaped sharp-angle furrow plow, which is capable of opening furrows with a width of 200 to 400 mm and a depth of 175 to 250 mm. The trapezoidal furrow has a stability of up to 90% in terms of width and depth. The effectiveness of the furrowing operations is in accordance with the desired outcome.

### 3.2 Structure and working principle of sowing depth adjustment device

The device for adjusting the depth of sowing is composed of three principal components: a parallel four-bar profiling mechanism, a depth-limiting wheel, and a furrow opener. During operation, the double-disc furrow openers performs the function of cutting the soil layer and pushing the soil to both sides in order to form the seed furrow. At the same time, there are synchronized depth limiting wheels on the left and right sides of the double disc opener, which can be used to adjust the initial depth of the furrow by means of the sowing depth adjustment handle. The seeds fall into the seed furrow from the seed tube, the suppression wheel then compacts the seeds and the soil, and finally the mulcher mulches the soil. During operation, the four-bar profiling mechanism enables the height of the furrow opener and the suppression wheel to be adjusted in accordance with the terrain. Modifications to the compaction and furrow depth can be accomplished by modifying the operational length of the spring and the elevation of the compaction wheel. The parallel four-bar profiling mechanism can ensure the constant soil entry angle of the opener, thereby enabling the opener to float up and down relative to its initial position. The depth-limiting wheel rolls in contact with the ground in real time, following the ups and downs of the ground surface. This ensures that the vertical distance between the wheel and the opener remains constant and unchanged, thereby determining the depth of the open furrow. The parallel four-bar profiling mechanism and the depth-limiting wheel work in conjunction to guarantee that the openers maintain a uniform depth of the seed furrow in undulating terrain. This ensures the consistency of cotton seedlings and crop growth, as well as the cleanliness of the conditions provided.

#### 3.2.1 Parallel four-bar profiling mechanism

**Figure 3:**
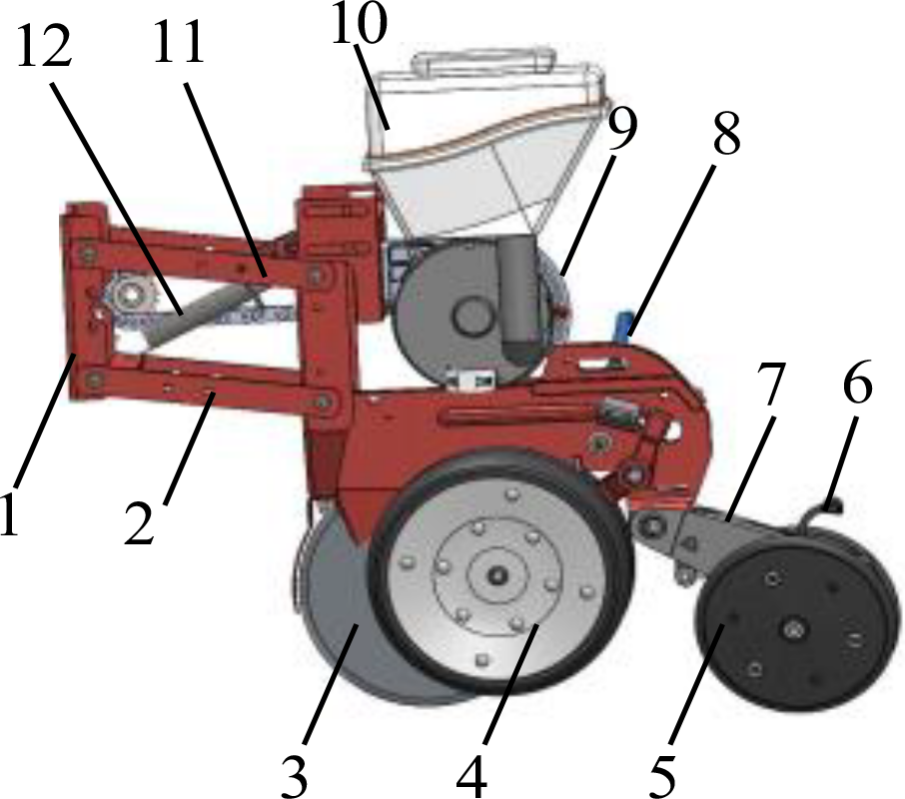
Schematic diagram of sowing depth control device. 1. frame connecting plate 2. lower tie rod 3. double disc opener 4. depth limiting wheel 5. suppression wheel 6. suppression wheel adjusting lever 7. suppression wheel connecting plate 8. depth limiting wheel adjusting lever 9. seed discharger 10. seed box 11. upper tie rod 12. adjusting spring.

**Figure 4:**
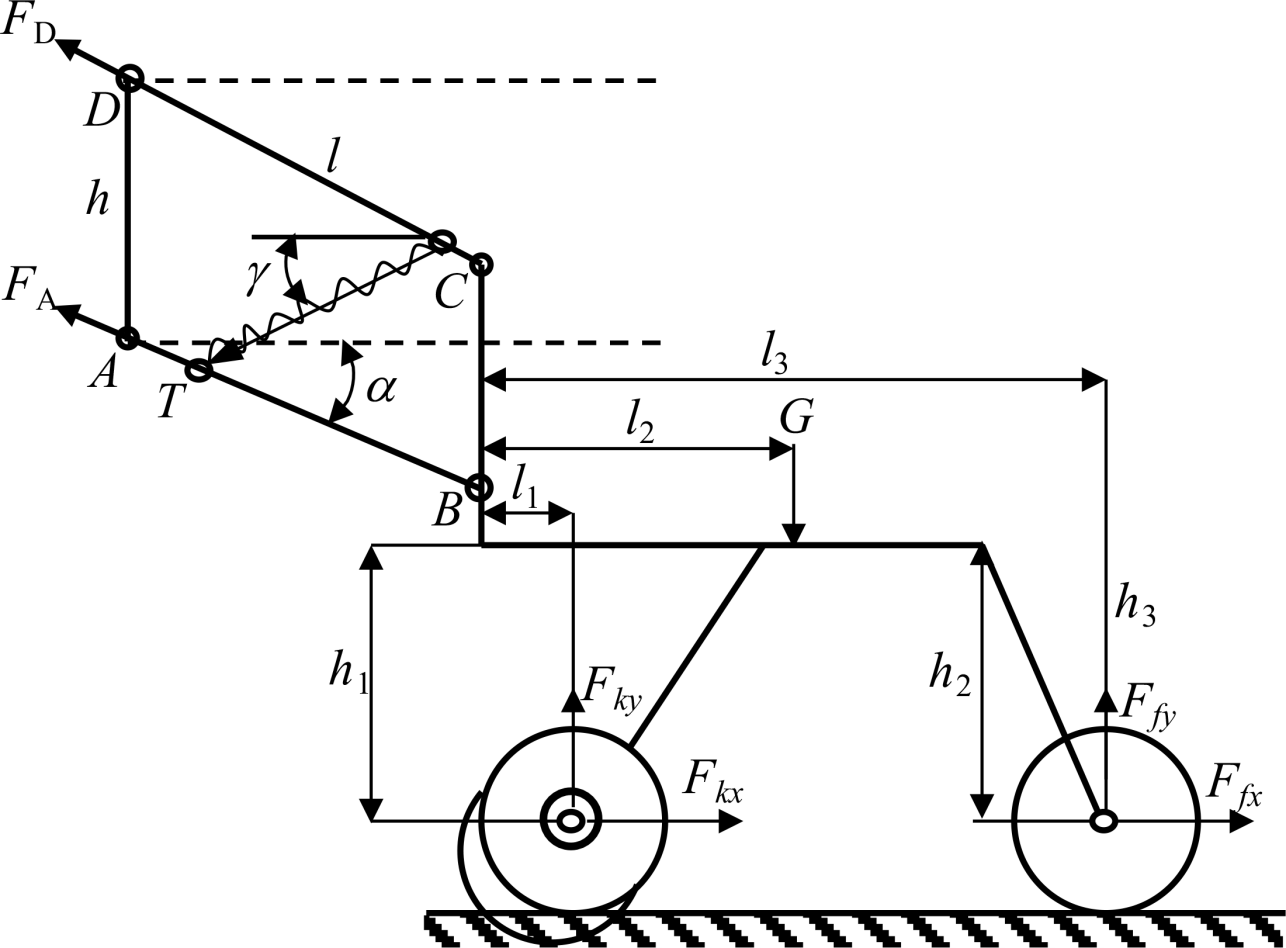
Schematic diagram of the structure of the sowing depth control device.

The inconsistency of furrowing depth has a significant impact on seedling emergence rate and crop yield. Therefore, it is of paramount importance to ensure the stability of furrowing depth. The four-bar imitation monobloc structure is straightforward and adaptable, and the imitation structure can guarantee that the furrowing monobloc changes in accordance with the undulation of the soil surface, thereby ensuring consistency in furrowing depth. The structure of the seeding monolith is shown in **Figure 3**. In this paper, the four-bar profiling mechanism is affixed to the frame and crossbeam via U-bolts. The upper and lower two connecting rods are of identical length and parallel distribution. The connecting rods are connected to the furrowing monobloc mounting plate via pins, which can be rotated around the pins. The axial direction is fixed by the sleeve. As a consequence of the variability in soil resistance and terrain undulation, the reaction force of the imitation wheel support also exhibits fluctuations. To ensure the stable operation of the furrow opener, the tension spring on the four link is adjusted so that the imitation wheel is maintained at a suitable grounding pressure, there by ensuring that the furrowing depth is essentially consistent. In the event that the soil is of a hard consistency, the quality of the soil is poor, or the weight of the furrowing monobloc is lighter than optimal, the tension or pressure spring on the four link is installed with the objective of increasing the grounding pressure of the imitation wheel. The furrowing monobloc is capable of floating up and down in conjunction with the movement of the upper and lower two links, thereby achieving a uniform furrowing depth. The supporting reaction force of the soil on the imitation wheel is dependent upon a number of factors, including the gravity of the entire machine, the resistance of the soil layer to the furrow opener, the working resistance of the pressure wheel, and the initial traction angle α of the imitation mechanism (Jing et al., 2022; Shang et al., 2024). A force analysis of the furrow opener monoblock in operation is presented in **Figure 4**.

The force equilibrium condition of the whole machine in stable operation is:


(1) $$\left\{ \begin{array}{l} F_{A}\cos\alpha +F_{D}\cos\alpha +T\cos\gamma -F_{kx}-F_{fx}=0\\ F_{A}\sin\alpha +F_{D}\sin\alpha -T\sin\gamma -G+F_{ky}+F_{fy}=0\\ F_{kx}=\mu F_{ky} \end{array}\right.$$

Where *F*_A_ is the force on the lower tie rod, N; *F*_D_ is the force on the upper tie rod, N; *F*_kx_ and F_ky_ are the horizontal and vertical components of the force on the depth-limiting wheel, N; *F*_fx_ and F_fy_ are the horizontal and vertical components of the force on the soil suppressor mulch, N; *α* is the initial traction angle of the profiling mechanism,°; *G* is the weight of the trenching monobloc, N; *l* is the length of the long parallel four-link bar, mm; *h* is the length of the short parallel four-link bar length, mm; *l*_1_ is the length of the four-bar mechanism to the point of the trench opener connecting rod, mm; *l*_2_ is the length of the four-bar mechanism to the center of mass of the monobloc, mm; *l*_3_ is the length of the center of mass of the overburden suppressor to the point of the center of mass of the four-bar mechanism, mm; and *u* is the damping coefficient of the trench opener.

The vertical reaction force on the furrow opener is obtained by collating Equation 1:


(2) $$F_{ky}=\frac{G+T(\sin\gamma +\cos\gamma \tan\alpha )-F_{fx}\tan\alpha -F_{fy}}{1+\mu \tan\alpha }$$

In order to ensure the stability of the planter’s furrowing operation under the undulating condition of the field surface, it is necessary to keep *F_ky_* > 0:


(3) $$\left\{ \begin{array}{l} T>\frac{F_{fy}+F_{fx}\tan\alpha -G}{\sin\gamma +\cos\gamma \tan\alpha }\\ T=k\Delta x \end{array}\right.$$

In uneven soil conditions, ensuring consistent planting depth requires an optimal spring preload. If the preload is too small, the downward force on the imitation mechanism is insufficient, resulting in shallow Sowing depth. Conversely, excessive preload causes the Sowing depth to be too deep. Therefore, with a fixed spring stiffness coefficient, the appropriate preload must be determined by calculating the initial spring deflection (Δ*x*).

Taking the moment at the four-link A as 0 yields:


(4) $$\begin{array}{c} F_{D}h\cos\alpha +F_{ky}(l\cos\alpha +l_{1})+F_{kx}(l\sin\alpha +h_{1})+F_{fx}(l\cos\alpha +l_{3})\\ +F_{fx}(l\sin\alpha +h_{2})-G(l_{2}+l\cos\alpha )-Tl_{AT}\sin(a+\gamma )=0 \end{array}$$

**Figure 5:**
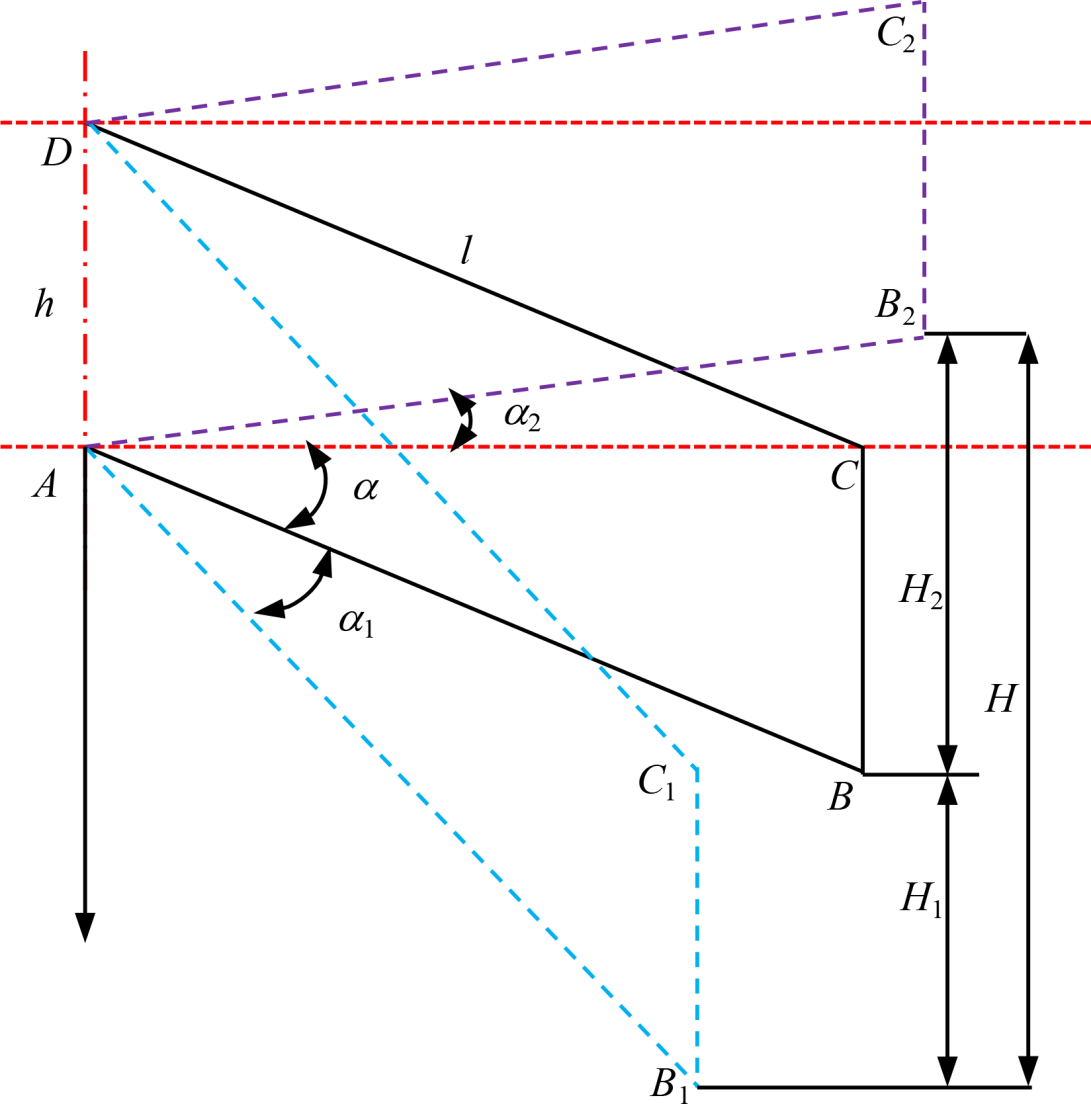
Schematic diagram of the parallelogram profile.

From Equation 4, it can be seen that in order to ensure the stable operation of double disc opener, and at the same time, it is necessary to consider the length l of the long rod of the imitation mechanism, the initial traction angle a. The parallelogram imitation structure is analyzed as shown in **Figure 5**.

The profiling volume H of the profiling mechanism is:


(5) $$\left\{ \begin{array}{l} H_{1}=l\sin(\alpha_{0}+\alpha_{1})-l\sin\alpha_{0}\\ H_{2}=l\sin(\alpha_{0}+\alpha_{2})\\ H=H_{1}+H_{2} \end{array}\right.$$

Where *H* is the total profiling distance of the profiling mechanism, mm; *H*_1_ is the profiling distance, mm; *H*_2_ is upper profiling distance, mm; *α*_1_ is upper profiling change angle, °; *α*_2_ is lower profiling change angle, °.

As illustrated in Equation 5, when the total imitation amount of the imitation mechanism is fixed, an increase in the length (l) of the parallel four-link rod results in a reduction in the total change angle of the imitation mechanism in the up-and-down direction. This is accompanied by an increase in the longitudinal size of the implement and a lowering and shifting back of the center of gravity of the entire machine. This configuration enhances the stability of the trench digger’s operation, which is beneficial for effective trenching. Although the length of the parallel four-link long rod (l) is larger, the center of gravity is shifted back, which is conducive to stable operation of the trenching machine. However, this results in a reduction in suspension stability of the entire machine. It is therefore essential to select an appropriate length of the parallel four-link long rod and the upper and lower profiling angle in order to ensure the same depth of open furrow in undulating terrain. The total change angle (*a_1_* + *a_2_)* must be less than or equal to 45 degrees in order to ensure general imitation.

A comprehensive consideration of the entire machine for opening the furrow and suspension operations was conducted under the premise of stability, normal operation of the double disc opener, and the design of the upper imitation change angle a2 = 10°, the lower imitation change angle *a*_1_ is 10°, the initial traction angle *a_0_* is 0, the length of the parallel four-link long rod 260mm, short rod 100mm. These values were then substituted into formula (5), resulting in a calculated value of H is 90mm. The general sowing machine imitation distance is typically within the range of 80-100mm, indicating that this design of imitation structure meets the specified design requirements.

#### 3.2.2 Synchronized limited depth double disc openers

**Figure 6:**
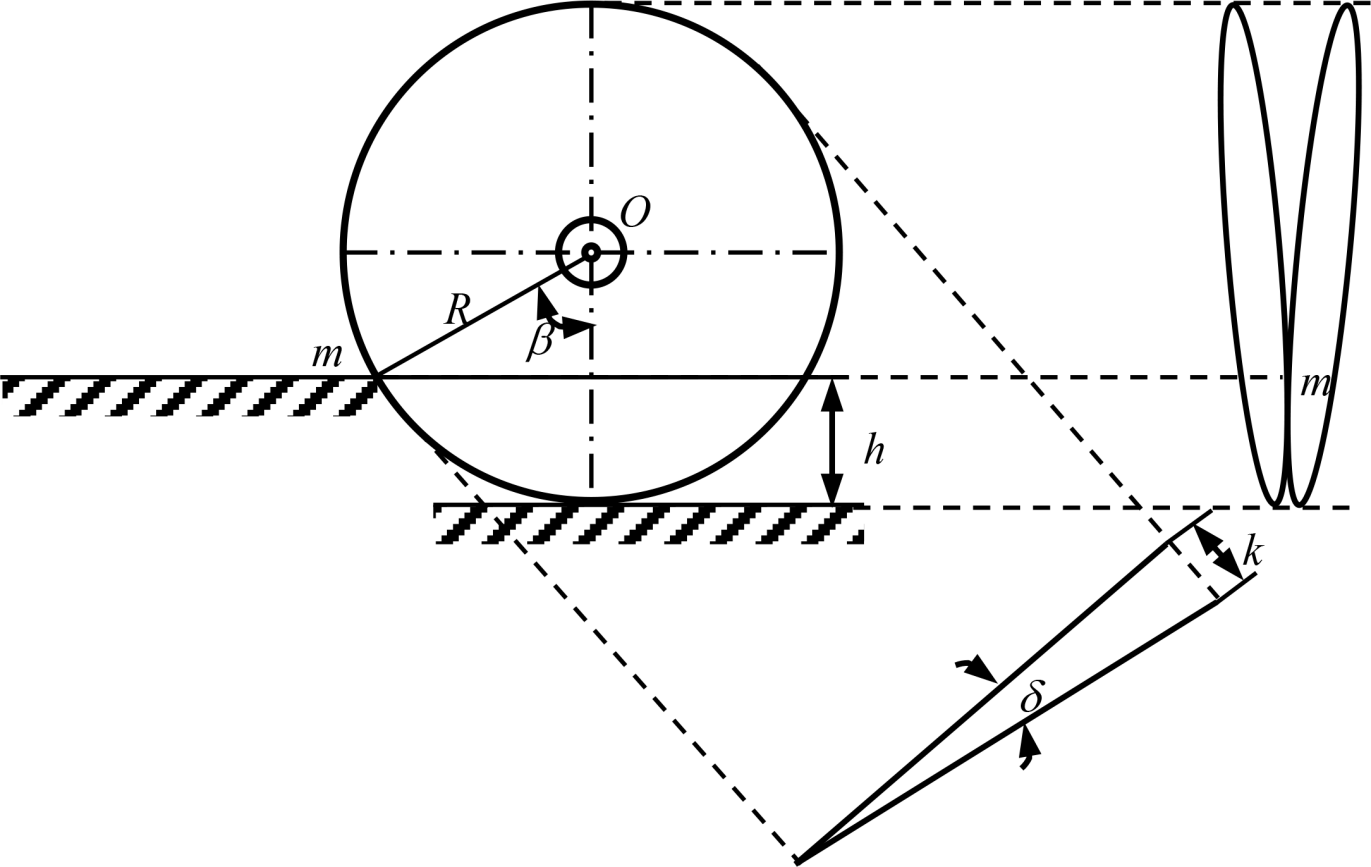
Double disc opener structure diagram.

The double disc opener functions by lifting, throwing, pushing, or squeezing a portion of the soil to create furrow marks. A schematic representation of the design and furrow profile is provided in **Figure 6**. The disc radius is insufficiently large, rendering the implement susceptible to rotation and jamming, which in turn increases resistance. The machine has been designed with a disc radius of *R* is 190 mm.

A reduction in disc angle (d) will result in a corresponding reduction in the size of the open trench and the working resistance. However, a value of *d* is 15 would result in the space between the two discs being insufficient to accommodate the seed guide tube. This paper presents a comprehensive consideration of the aforementioned issue.


(6) $$\left\{ \begin{array}{l} \cos\beta =\frac{R-h}{R}\\ \text{ > }L=2R\sin\beta \\ \;K=2L\sin\frac{\delta }{2} \end{array}\right.$$

Where *β* is the angle of poly point m in the vertical direction, °; *d* is the angle of disc, °; *R* is the radius of disc, mm; *h* is the depth of trenching, mm; *L* is the corresponding chord length of the position of the poly point, mm; *K* is the width of trenching, mm.

In accordance with the furrow depth of cotton direct seeding, which ranges from 30 to 40mm, this paper selects 40mm as the maximum cutting depth of the furrow opener in the soil cutting process. The position of the polygonal point is expressed by the β angle, which is indicative of the width of the furrow. A larger *β* angle corresponds to a wider furrow, while a smaller β angle results in a narrower furrow. Additionally, *β* angle that is too small may cause the m point to be positioned too low, leading to soil from the top of the polygonal point entering between the double discs. This can result in the disc clamping soil and clogging. Substitution of the aforementioned disc parameters into Equation 6 yields the disc groove width *K*=60.89mm and *β*=37.86°. It is capable of meeting the requisite specifications for cotton operations within the Yangtze River basin. Additionally, synchronized depth-limiting wheels are integrated into the design of the double disc opener, positioned on both the left and right sides. These wheels facilitate the adjustment of the furrow depth through the sowing depth adjustment handle, ensuring uniform seed depth. This innovative approach effectively addresses the limitations of traditional depth-limiting structures. The depth-limiting wheel is designed with a width of 120 mm, a diameter of 385 mm, and six gears. A single adjustment of the depth of the furrow results in a change of 10 mm. The inner circle remains in close proximity to the disc, thereby preventing clogging. To prevent the soil from adhering to the outer edge of the depth-limiting wheel and resulting in inconsistent furrowing depth, the outer end of the wheel is designed with a scraper.

### 3.3 Seeding control system and device

#### 3.3.1 Spoon tooth cotton precision seed displacer

**Figure 7:**
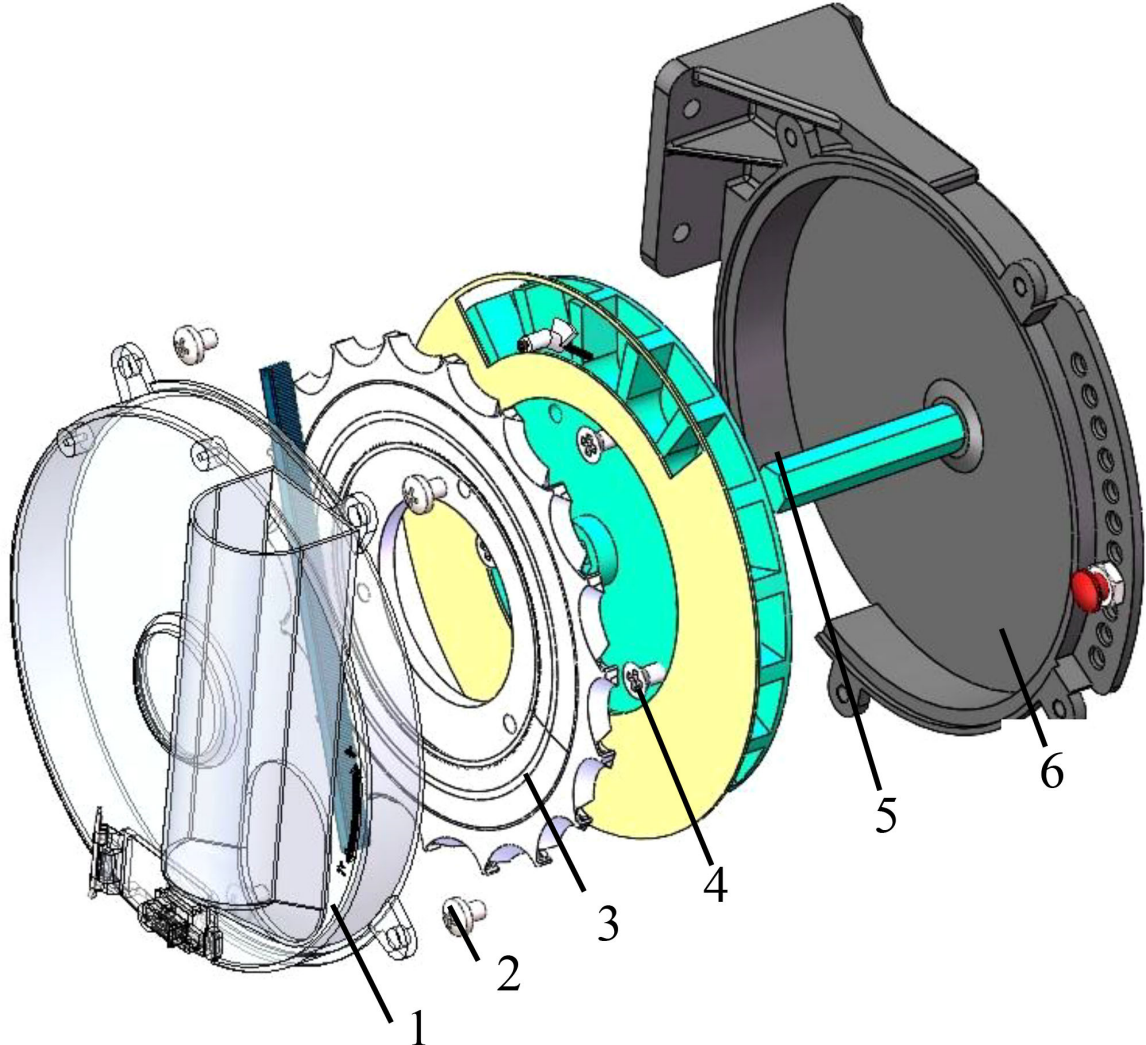
Seed discharger structure. 1. Left housing 2. Screw 3. Spoon tooth type seed discharging disk 4. Seed dividing disk 5. Seed discharging shaft 6. Right housing.

The spoon tooth type cotton precision seed discharger is comprised of several key components, including a left shell, screws, a spoon tooth-type seed discharging disk, a seed dividing disk, a seed discharging shaft, and a right shell. The structure is illustrated in **Figure 7**. The operational mechanism of the seed discharger can be delineated as such: cotton seeds initially enter the left shell seed chamber through the seed box. The seed discharger is powered by a driving direct current (DC) motor, which enables it to rotate at an optimal velocity. The cotton seeds are conveyed by the teeth of the seed spoon of the seed discharging disk. The seed discharging disk and the ring-shaped seeding disk are held captive to the cotton seeds. When the seed spoon carries more than The excess seeds are then removed by the seed clearing brushes. As the seed discharging disk rotates to the gap at the upper end of the ring-shaped seeding disk, the seeds fall into the seed cavity wheel chamber. The seedcavity wheel and the seed discharge disk are connected by bolts to realize synchronous rotation. When the seed cavity carrying the seeds moves to the seed casting mouth, the cotton seed discharge operation is realized.

**Figure 8:**
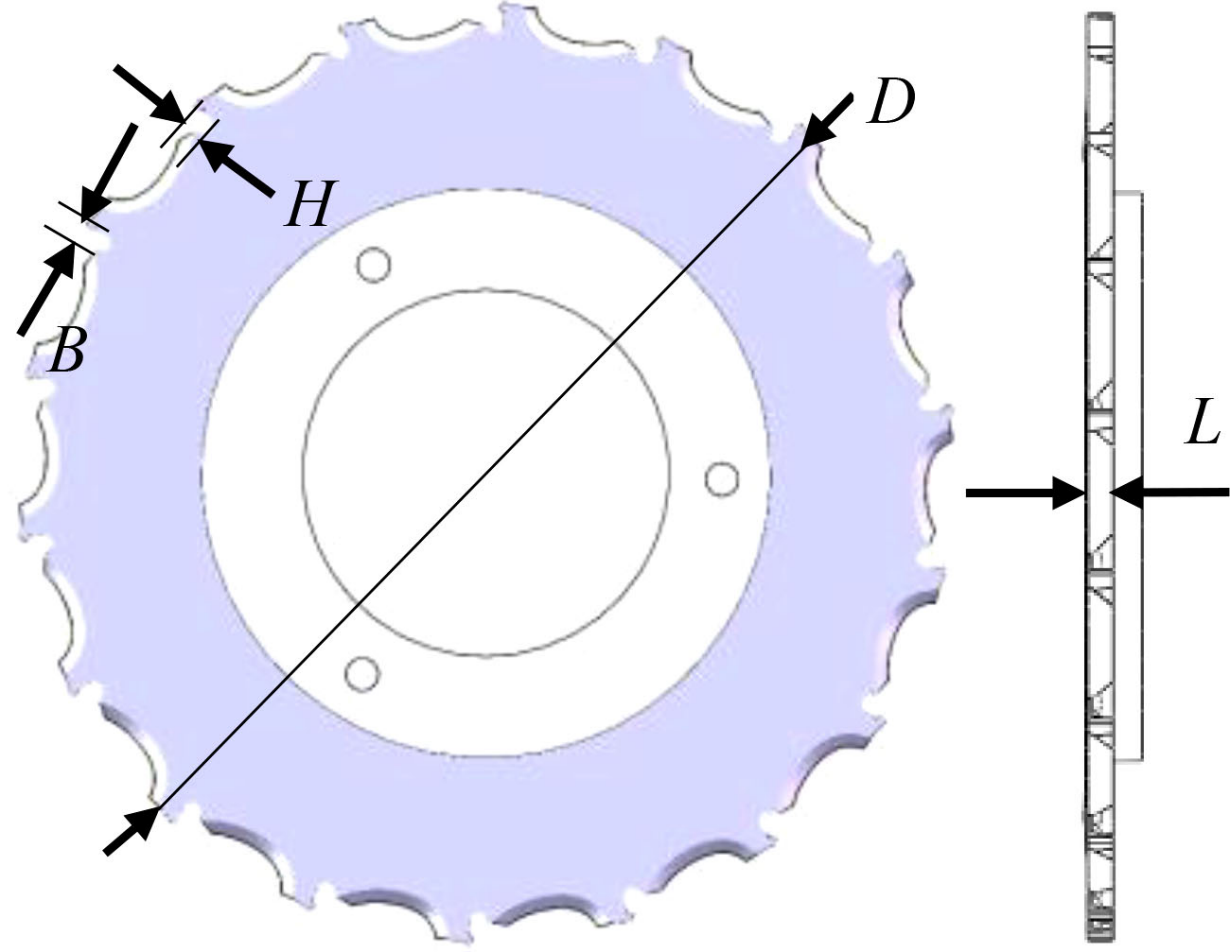
Structural diagram of spoon-tooth seed tray.

Cotton seed class ellipsoid shape, selected 100 30 cotton seeds, measured the average triaxial dimensions of the seed: length *l_0_* = 9.10mm, width *b_0_* = 4.88mm, thickness *h_0_* = 4.18mm. from the review of the literature, the general diameter of the hole-type seed tray 80 ~ 260mm, this paper selects 230mm, the center of the seed hole distance of 218mm. The structure of the seed discharge disk type hole is shown in **Figure 8**.

Under the determination of the diameter of the seed tray, the fewer the number of seed holes, the more conducive to seed filling, seed filling effect is good; but the fewer the number of seed holes, the number of planting units of the planter is less than the planting agronomic requirements. Therefore, the number of seed holes should meet the following formula:


(7) $$z=\frac{60\cdot v_{m}}{S\cdot n_{p}}$$

Where *v_m_* is the Working speed of the opener, m/s; *S* is the plant spacing, m; *n*_p_ is the rotational speed of the seed discharge disk, r/min; *Z* is the number of seed holes. In this paper, the design of the seeding machine Working speed of 0.8~1.7m/s, row of seed disk speed ≤ 45r/min, plant spacing 14.6-21.9cm between the adjustment; here take the furrowing machine Working speed of 1.5m/s, row of seed disk speed of 35r/min, plant spacing of 15cm; to get the number of holes in the row of seed disk to take the seeds and rounded up to 18.

Design principles to be followed for the structural dimensions of seed extraction holes according to the requirements of precision seed extraction:


(8) $$\left\{ \begin{array}{l} L=l_{0}+(1∼1.5)\\ B=h_{0}+(0.7∼1)\\ \frac{1}{3}l_{\max}\leq H\leq 1.2b_{0} \end{array}\right.$$

Where *l*_0_ cotton seed length, mm; *b*_0_ is cotton seed width, mm; *h*_0_ is cotton seed thickness, mm.

According to the above formula (cotton seed size parameters, the average length of the coated cotton seed *l_0_* is 9.10mm, the maximum length *l*_max_ is 10.77mm, the average width *b*_0_ is 4.88mm, and the average thickness of *h*_0_ is 4.18mm), the length of the seed hole is taken as *L* = 10.5, the width of the hole is *B* = 5.18, and the thickness of the hole is *H* = 4.5.

#### 3.3.2 Seeding control system

**Figure 9:**
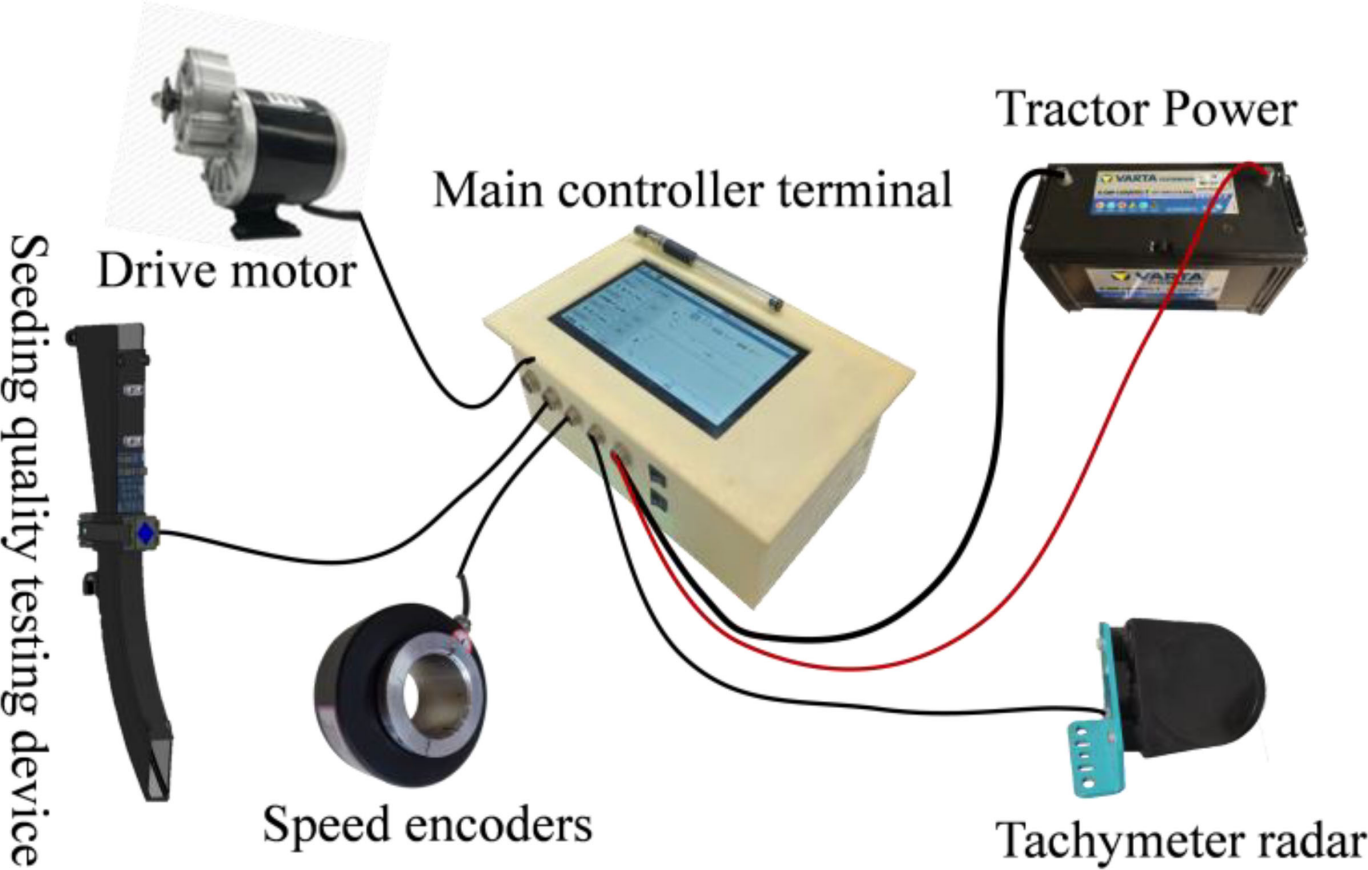
Structure diagram of variable-rate seeding control system.

The desired seed spacing can be maintained only when the RPM of the seed metering plate is regulated according to the forward speed of the tractor (Mahapatra et al., 2024b). The traditional cotton precision direct seeding machine is designed to utilize a passive ground wheel drive seed discharge device, open furrow, and operate in complex working conditions. However, the ground wheel is prone to slippage, which can result in seed leakage, broken strips, and other undesirable outcomes. This can negatively impact the precision of furrowing operations. Additionally, manual transmission adjustment of seeding is challenging, making it difficult to achieve precise adjustments to furrowing grain spacing, seeding volume, and other crucial parameters. To address these limitations, a Raspberry Pi has been proposed as the primary controller for cotton precision direct seeding machines. This integration aims to enhance the control of furrowing operations, ensuring more precise and efficient seed placement and distribution. The control system hardware is shown in **Figure 9**. The primary control unit of the cotton furrowing machine, which oversees the speed of the furrowing control system, is comprised of an information acquisition module, a speed controller, and a series of seed actuators. The information acquisition module incorporates a speed radar and high-precision coding. The seeding actuator comprises a cotton precision seeder, a DC geared motor, and a coupling. The hardware component of the speed of furrow controller encompasses modules such as a DC motor driver, a touchable LCD, a Raspberry Pi 4B, a built-in sub power supply, and an IC. The software component of the high-speed furrow controller is founded upon the examination of multithreading technology within the Linux Qt framework. This encompasses the development of a signal reception and calculation thread, a control algorithm processing thread, and a graphical interface and touch interaction thread. These threads collectively facilitate the reception and analysis of signals, the implementation of algorithmic code, and the interaction between control, interface display, and touch functions. The development language is C++, and the software development framework is Linux Qt. The integrated development environment (IDE) is Qt Creator 4.8.0.

**Figure 10:**
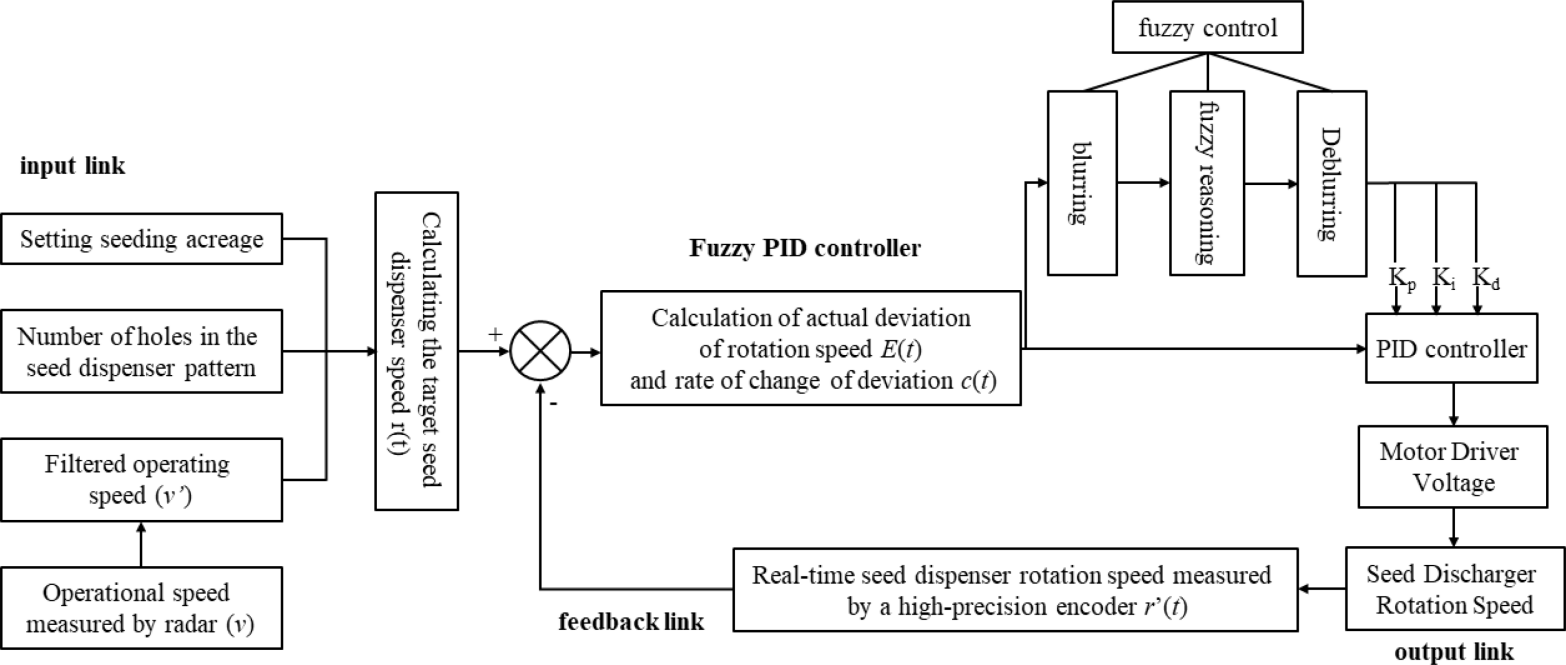
Program flow chart of seeding control system.

Program flow chart of seeding control system as shown in **Figure 10**. The operational principle of the system is as follows: upon initiation of the planter, the Raspberry Pi control terminal receives the input of the desired seeding rate or plant spacing and calculates the requisite seeding demand to achieve the current seeding rate or plant spacing, taking into account the forward speed of the planter, which is measured by the speed radar. The system then determines the target seeding speed that the planter must achieve. The high-precision encoder measures the current speed of the planter and serves as a feedback mechanism. The Raspberry Pi control terminal then compares the current speed of the planter with the target speed, as indicated by the feedback from the high-precision encoder. Based on this comparison, the terminal sends a calibration signal to the driver. This signal is generated after the system control algorithm is calculated, allowing for the adjustment of the actual rowing speed of the planter. The PID control algorithm then regulates the actual rowing speed, resulting in the accurate speed control of the planter. The control system utilizes the Raspberry Pi 4B as the primary control chip. The human-computer interactive graphical user interface (GUI) display interface configures the system furrowing operation mode and parameters. The speed radar and high-precision encoder obtain the Working speed and rotational speed of the seed discharger shaft, respectively. Based on the fuzzy PID algorithm, the system then adjusts its output, and the seed discharger is driven by the direct-current (DC) geared motor to discharge the seed, thereby achieving a closed-loop speed control and precise adjustment of cotton furrowing operations. Prior to initiating field operations, the operator is afforded the option of manually modifying the system setup parameters via the touchable graphical user interface (GUI) of the controller. The display shows real-time information about the current planter Working speed, seed dispenser shaft speed, and the amount of seed that has been planted.

## 4 Bench test

### 4.1 Test materials and equipment

**Figure 11:**
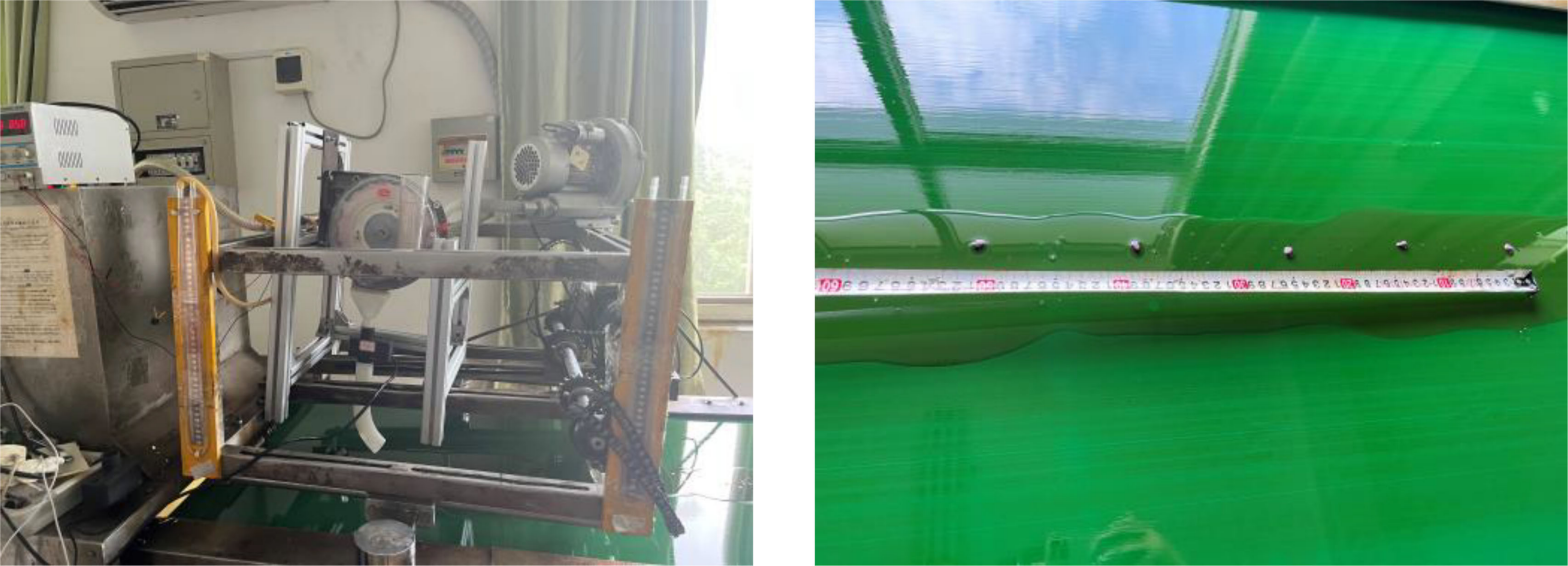
Seed metering test bed.

To test the seed discharging performance of the designed spoon tooth type cotton precision seed discharger, the seed discharger was installed on the JPS-12 model computer vision seed discharging performance test bench as shown in **Figure 11**. The test material was selected from the commercialized seed of Ewa cotton 30, which was widely planted in the Yangtze River basin, with a water content of 8.65%, a thousand grain mass of 91.8g, and a sphericity of 63%.

### 4.2 Bench test results

**Figure 12:**
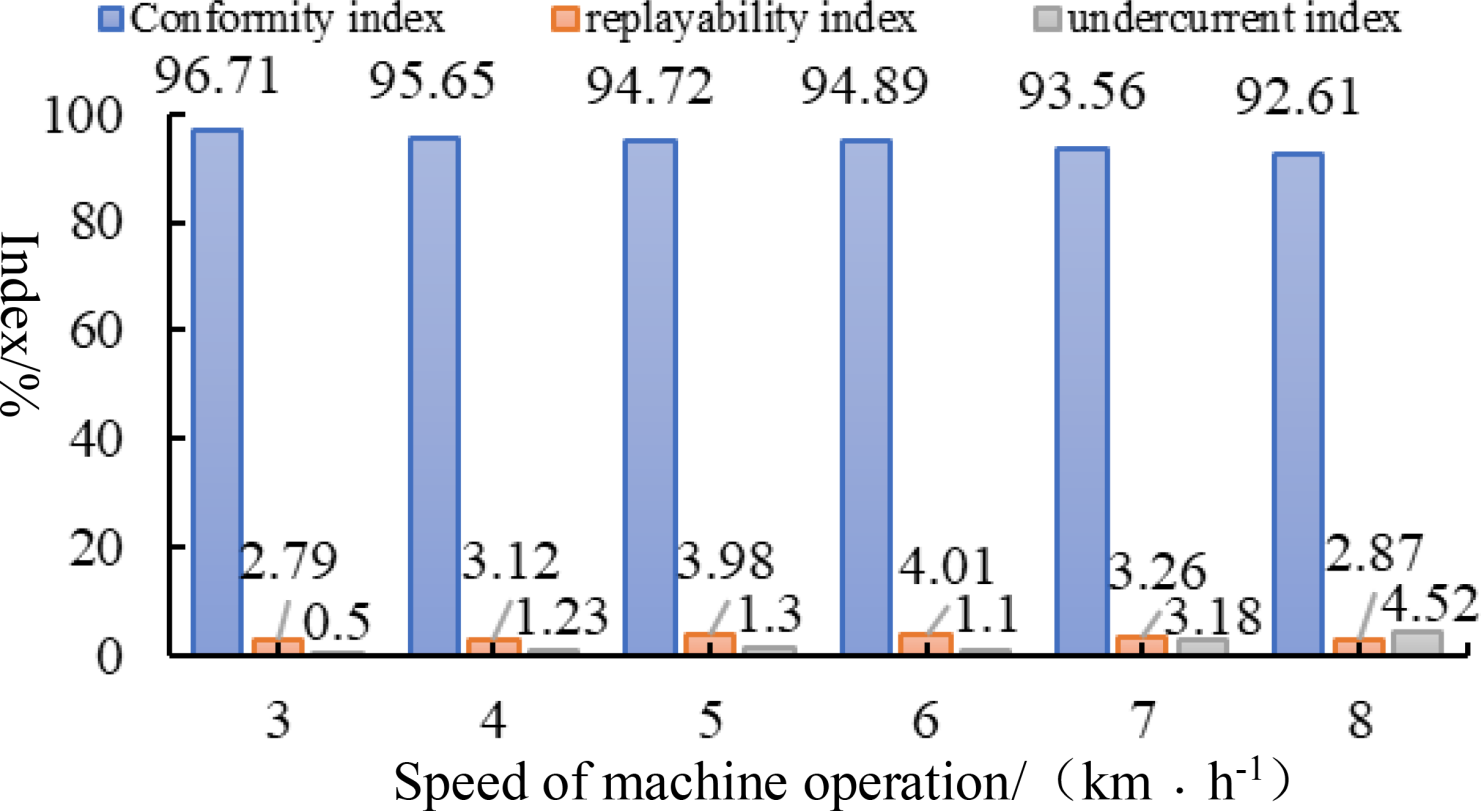
Test results.

In accordance with the test methods set forth in GB/T6973-2005 for single grain (China Precision) openers, at a working speed of 3-8km·h^−1^, with plant spacing set at 10cm, the working speed serves as the test condition, while the conformity index, replayabiltity index, and undercurrent index represent the evaluation indexes. Each group of tests is repeated three times, and the average value is taken. The results of the aforementioned tests are illustrated in **Figure 12**. As the Working speed increases from 3 to 8 km·h^−1^, the overall conformity index of the seed discharger declines. However, the overall conformity index remains above 90%, and the conformity index exhibits minimal variation. Consequently, the overall conformity index is sufficient to meet the design specifications of the furrow opener. As the Working speed exceeds 6 km·h^−1^, the leakage sowing index increases. This is due to the fact that as the rotational speed of the seed discharging disk rises, the seed taking hole charging time is reduced, leading to an intensification of the leakage sowing phenomenon. However, at an Working speed of 8 km·h^−1^, the undercurrent index remains below 5%, demonstrating that the seed discharger can still fulfill the requirements of cotton precision furrowing at speeds between 3-8 km·h^−1^.

## 5 Field trial

### 5.1 Test condition

The test site is located in the experimental base of Huazhong Agricultural University in Anlu City, Hubei Province, which is located in the middle and lower reaches of the Yangtze River Basin, and the soil type is yellow-brown loam with medium soil fertility. The test results show that the soil moisture content is 42.3%, the moisture content is suitable, pH 6.83, neutral soil, the soil moisture content and pH in this area to meet the needs of cotton seed growth and development. The supporting power was Case TM1404 tractor.

### 5.2 Stability of trenching depth

#### 5.2.1 Evaluation index of trenching depth stability

According to the agronomic requirements of cotton cultivation furrow depth 3~4cm, combined with the agronomic requirements, set the theoretical value of cotton furrow depth 3cm, this test cotton furrow depth is qualified determination standard for h ± 1.0cm, that is, the depth of the furrow between 2~4cm is qualified. Using the initial traction angle, spring steel, equipment Working speed as the test factors, furrow depth conformity index, furrow depth coefficient of variation as the test index, to carry out the second orthogonal rotary combination test, each group of tests repeated three times, calculate the average value of the measurement results. The length of the test plot was 10m, and 5m test points were selected in each row and evenly distributed within the test plot. At the test point, the soil layer is cut vertically, 10 cross-sections are taken at equal intervals, and the vertical distance from the original surface to the bottom of the trench is measured as the trench depth. The rooting depth qualification index is calculated as follows:


(9) $$H=\frac{h_{1}}{n}\times 100\%$$

where *H* is the conformity index of open furrow depth, %; *h*_1_ is the number of holes with qualified open furrow depth; *n* is the total number of holes in the test area.

The coefficient of variation of trenching depth consistency is calculated as:


(10) $$V_{h}=\frac{\sqrt{n\times \sum_{i=1}^{n}{\left(h_{i}-\frac{\sum_{i=1}^{n}h_{i}}{n}\right)}^{2}}}{\sum_{i=1}^{n}h_{i}}\times 100\%$$

Where *V_h_* is the coefficient of variation of trench depth consistency; *h*_i_ is the depth of the trench at the measurement point, mm.

#### 5.2.2 Analysis of the results of the open furrow depth field experiment

From the above analysis, the initial pull angle α, the initial spring increment *Δ_x_* and the working speed have a great influence on the trenching depth, and the initial pull angle, the initial spring increment and the working speed of the machine were taken as the experimental factors, and the levels of the factors ranged from -10° to 10°for the initial pull angle, 10-25 mm for the initial spring increment and 3-7 km·h^-1^ for the working speed of the trencher. The factor coding table is shown in **Table 2**. In the process of field testing, the furrow opener can be adjusted according to different actual conditions. Through the test, the digging depth qualification index (*Y*), digging depth consistency coefficient of variation (*V*) were analyzed for significance, and the combination of each parameter was optimized according to the actual needs.

**Table 2:** Experimental factors and levels.

Level	Initial traction angle *X*_1_/(°)	Initial spring extension *X*_2_/mm	Working speed *X*_3_/(km·h^-1^)
-1	-10	10.0	3.
0	0	17.5	5
1	10	25.0	7

#### 5.2.3 Test results and analysis

The design of the test program was carried out according to the response surface method in Design-Expert 8.0.6 software, and the test program and results are shown in **Table 3**.

**Table 3:** Test scheme and result.

No.	*X*_1_	*X*_2_	*X*_3_	Ditch depth qualification index *Y*/%	Depth variation coefficient *V*/%
1	1	0	-1	93.5	8.2
2	0	0	0	91.8	9.3
3	0	1	1	74.5	10.5
4	-1	0	1	71.4	11.3
5	0	0	0	93.5	8.8
6	1	1	0	85.7	9.4
7	0	0	0	91.9	9.1
8	0	-1	1	74.6	13.6
9	0.	0	0	92.6	8.8
10	-1	1	0	82.5	8.5
11	0	0	0	92.7	9.2
12	-1	0	-1	90.3	7.5
13	1	-1	0	83.4	11.3
14	0	0	0	91.5	8.9
15	1	0	1	74.2	12.3
16	0	1	-1	93.0	8.4
17	-1	-1	0	81.7	9.3
18	0	-1	-1	88.4	8.7

The analysis of variance for the trenching depth qualification index is shown in **Table 4**, and a binary multiple regression was fitted to the data in **Table 3** to obtain the regression equation for the trenching depth qualification index, *Y*, on the respective variables as:

**Table 4:** Analysis of variance for the qualification rate of sowing depth.

Variation source	Sum of squares	d*f*	Mean square	F value	P value
Model	802.85	9	89.21	116.97	<0.0001**
*X_1_*	7.03	1	7.03	9.22	0.0162*
*X_2_*	9.68	1	9.68	12.69	0.0074**
*X_3_*	399.03	1	399.03	523.25	<0.0001**
*X_1_X_2_*	11.56	1	11.56	15.16	0.0046**
*X_1_X_3_*	14.82	1	14.82	19.44	0.0023**
*X_2_X_3_*	10.89	1	10.89	14.28	0.0054**
*X_1_^2^*	131.00	1	131.00	171.78	<0.0001**
*X_2_> ^2^*	53.58	1	53.58	70.26	<0.0001**
*X_3_^2^*	103.88	1	103.88	136.22	<0.0001**
Lack of fit	2.31	3	0.77	1.02	0.4586
Pure error	3.79	5	0.76		
Correct total	808.95	17			

*indicates that the item is significant (P<0.05); **indicates that the item is highly significant (P<0.01). Same as below.


(11) $$\begin{array}{c} Y=47.87-0.97X_{1}+2.88X_{2}+10.59X_{3}+0.23X_{1}X_{2}\\ +0.10X_{1}X_{3}-0.11X_{2}X_{3}-0.05X_{1}^{2}-0.06X_{2}^{2}-1.22X_{3}^{2} \end{array}$$

From the analysis of variance in **Table 4**, it can be seen that except for the influencing factors Lack of fit, which are not significant, the other influencing factors are extremely significant. The significance of each factor of the conformity index of trenching depth in descending order is the Working speed, initial traction angle, and initial increment of spring.

The analysis of variance of the coefficient of variation of trenching depth is shown in **Table 5**, and a binary multiple regression of the coefficient of variation of trenching depth consistency was fitted to the data in **Table 2** to obtain the regression equation of the coefficient of variation of trenching depth consistency to the respective variable as:

**Table 5:** Analysis of variance of the coefficient of variation of sowing depth.

Variation source	Sum of squares	d*f*	Mean square	F value	P value
Model	41.53	9	4.61	64.68	<0.0001**
*X*_1_	2.65	1	2.65	37.07	0.0003**
*X*_2_	4.65	1	4.65	65.19	<0.0001**
*X*_3_	27.75	1	27.75	388.92	<0.0001**
*X*_1_ *X*_2_	0.30	1	0.30	4.24	0.0735
*X*_1_ *X*_3_	0.023	1	0.023	0.32	0.5898
*X*_2_ *X*_3_	1.96	1	1.96	27.47	0.0008**
*X*_1_^2^	0.019	1	0.019	0.27	0.6162
*X*_2_^2^	1.28	1	1.28	17.94	0.0029**
*X*_3_^2^	2.40	1	2.40	33.64	0.0004**
Lack of fit	0.34	3	0.11	2.50	0.1739
Pure error	0.23	5	0.046		
Correct total	42.11	17			


(12) $$\begin{array}{c} V=9.64+0.10X_{1}-0.21X_{2}-0.11X_{3}-3.67\times 10^{-3}X_{1}X_{2}\\ +3.75\times 10^{-3}X_{1}X_{3}-0.05X_{2}X_{3}+6.67\times 10^{-4}X_{1}^{2}+9.63\\ \times 10^{-3}X_{2}^{2}+0.19X_{3}^{2} \end{array}$$

From the analysis of variance in **Table 5**, it can be seen that all the influence factors are significant except for the influencing factors *X*_1_ *X*_2_, *X*_1_ *X*_3_ and *X*_1_^2^, which are not significant. The significance of the coefficients of variation of trenching depth for each factor in descending order were Working speed, initial spring increment, and initial traction angle.

#### 5.2.4 Optimization of test results

**Figure 13:**
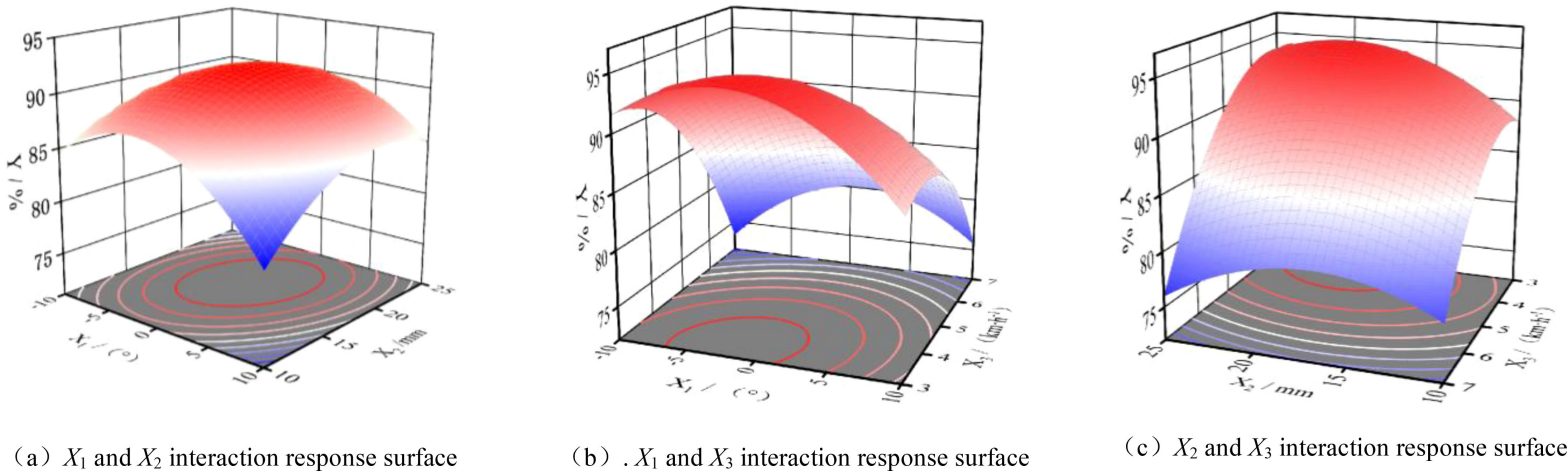
Response surface of the interaction of various factors on root-soil separation rate. **(A)** *X*_1_ and *X*_2_ interaction response surface. **(B)**. *X*_1_ and *X*_3_ interaction response surface. **(C)** *X*_2_ and *X*_3_ interaction response surface.

**Figure 14:**
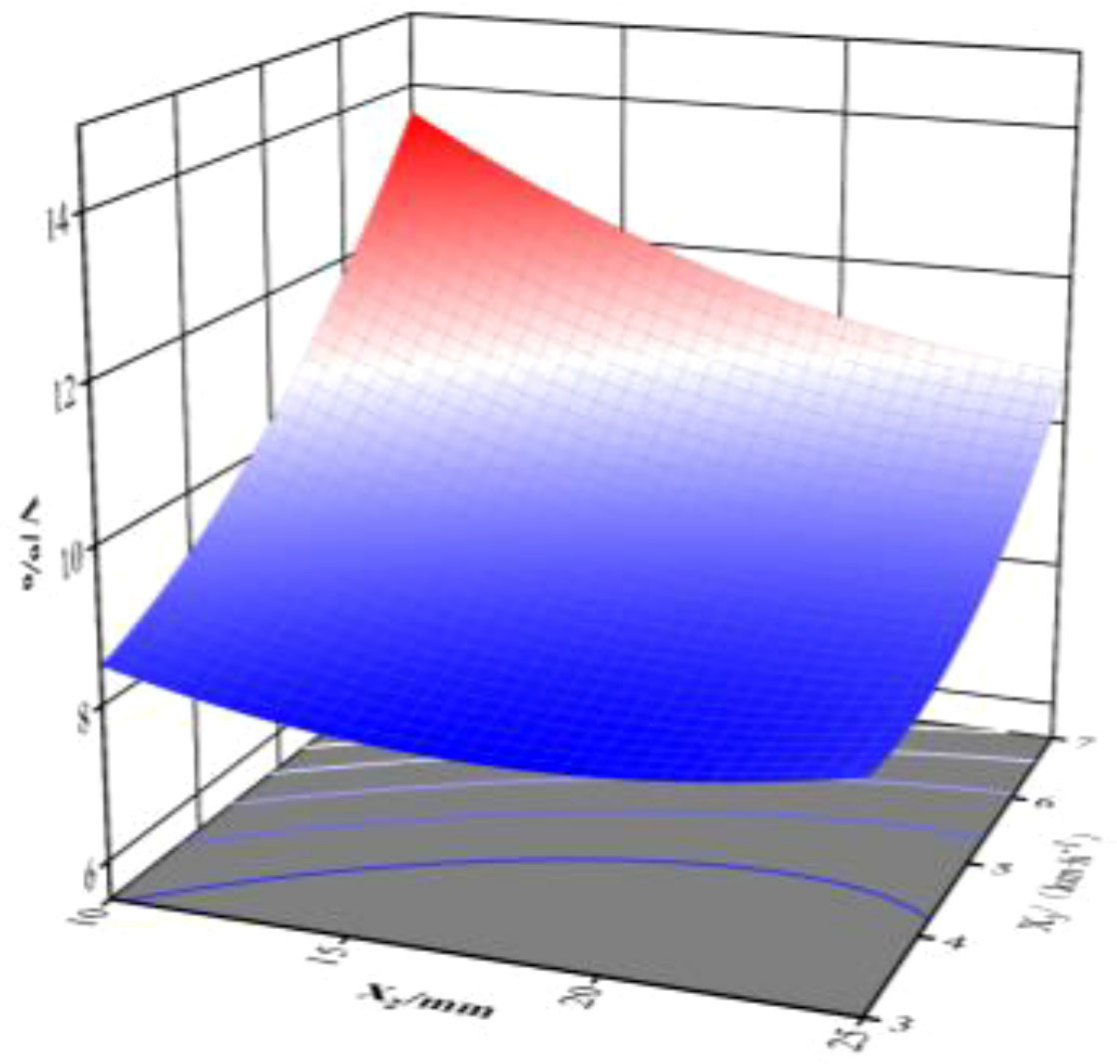
Response surface of double parameters about coefficient of ditch depth variation coefficient.

The response surface of the interaction between the initial traction angle, spring steel degree, and machine operating speed on the test index of furrowing depth qualification index was derived through data processing with the Design Expert 8.0.6 software, as illustrated in **Figure 13**. As illustrated in **Figures 14A, B**, an increase in the initial traction angle initially resulted in an increase in the qualified index of furrowing depth, followed by a subsequent decrease. The optimum initial traction angle range was determined to be -1.96 to 1. The optimal initial traction angle was found to be 54°, with the best initial traction angle being 0°. This indicates that the initial traction angle of the parallel four-link profiling structure is closer to the level of the favorable depth of the qualified index of furrowing depth. Furthermore, it suggests that the profiling monobloc has a small profiling oscillation in order to maintain the best furrowing depth. As the initial traction angle is fixed, an increase in the initial spring increment results in an initial rise in the furrow depth qualification index, which subsequently declines. The optimal spring initial increment is found to be within the range of 16.89 to 19.19 mm. This suggests that the spring should not be excessively large in terms of its initial preload on the furrow single body. A preload that is too large will result in a compression force on the seed bed that is too strong, leading to an excessive depth of furrow. As illustrated in **Figures 14B, C**, when the initial traction angle and the initial spring increment are held constant, the qualified index of furrowing depth declines with an increase in working speed. This suggests that an elevated working speed increases the furrowing depth of the furrowing monobloc due to the inertia of the system and other external disturbances.

When the initial traction angle is 0, the interaction between the initial increment of the spring and the operating speed of the implement responds to its response surface for the furrowing depth consistency coefficient of variation, as illustrated in **Figure 14**. At an operating speed of 5 km/h, the coefficient of variation of trenching depth consistency tends to decrease as the initial spring increment increases.

#### 5.2.5 Optimal parameter solution and field test validation

The optimization module in Design-Expert 8.0.6 software was utilized to solve the regression model, thereby determining the constraints of the test factors based on the actual working conditions and the aforementioned relevant model analysis results. The initial traction angle was determined to be −1.96 to 1.54°, the initial increment of the spring of 16.89 to 19.19 mm, and the operating speed of 3.0 to 4.0 km·h^-1^.The test indexes included the maximum value of the sowing depth qualification index and the minimum value of the sowing depth consistency coefficient of variation. The parameter optimization results yielded the following values: initial traction angle −1.60°, spring initial increment 19.03mm, operating speed 3.57 km·h^-1^. For the convenience of processing and testing, the initial traction angle was set to 0°, the spring initial increment to 20mm, and the operating speed to 3.6 km·h^-1^. The results showed that under the conditions of initial traction angle of 0°, initial spring increment of 20 mm and working speed of 3.6 km/h, the qualified rate of ditching depth was more than 85%; and the variation coefficient of ditching depth was less than 12%.

### 5.3 Field test for uniformity of seed placement

#### 5.3.1 Seed displacer speed control performance

**Figure 15:**
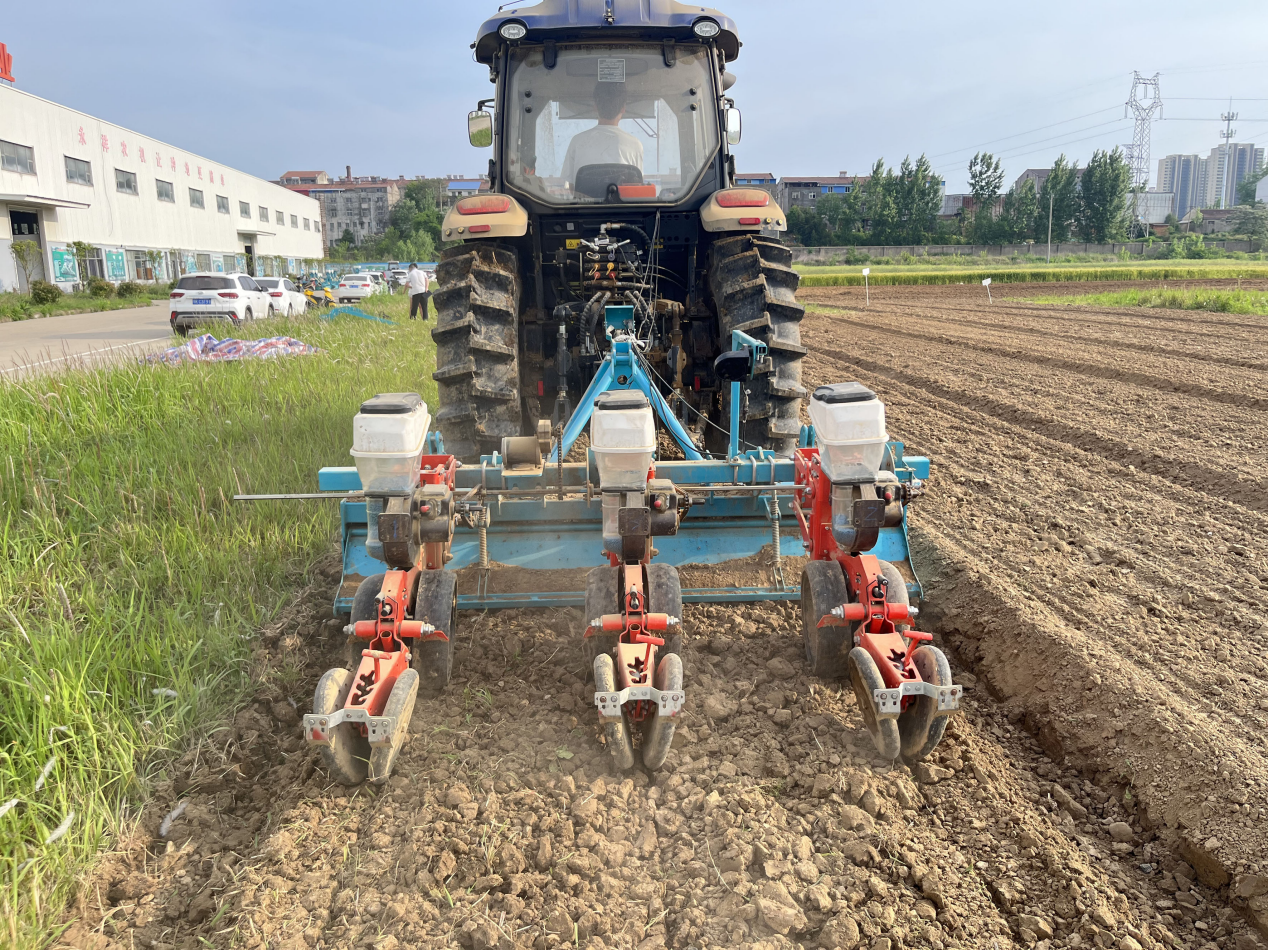
Seeding depth test site.

The field test of the seeding control with speed is shown in **Figure 15**. The speed of the seeder drive motor is measured using a Japan OMRON E6B2-CWZ6C600P/R incremental encoder. The output comprises three phases (A, B, Z) and is capable of supporting analog voltage signals. It is designed to rotate a circle and output 600 pulse signals, with a current of 70mA or less. The encoder is characterized by its small power consumption and strong anti-interference capabilities. In order to obtain feedback regarding the speed of the system, the A phase is connected to the signal transmission board interface. The Raspberry Pi GPIO is then used to read the pulse and calculate the current speed. The speed control system can be set to drive at a fixed speed and follow the speed of the two drive modes. The entire apparatus employs a direct current (DC) motor to drive three seed dischargers via an intermediate chain. The ratio of the chain transmission is 1:1, which aligns with the seed discharger seed discharge drive relationship. In normal operation, the effective speed range of the seed discharging disk is 5.10-45.90 rpm, with an input voltage range of 1.5-12V.

**Figure 16:**
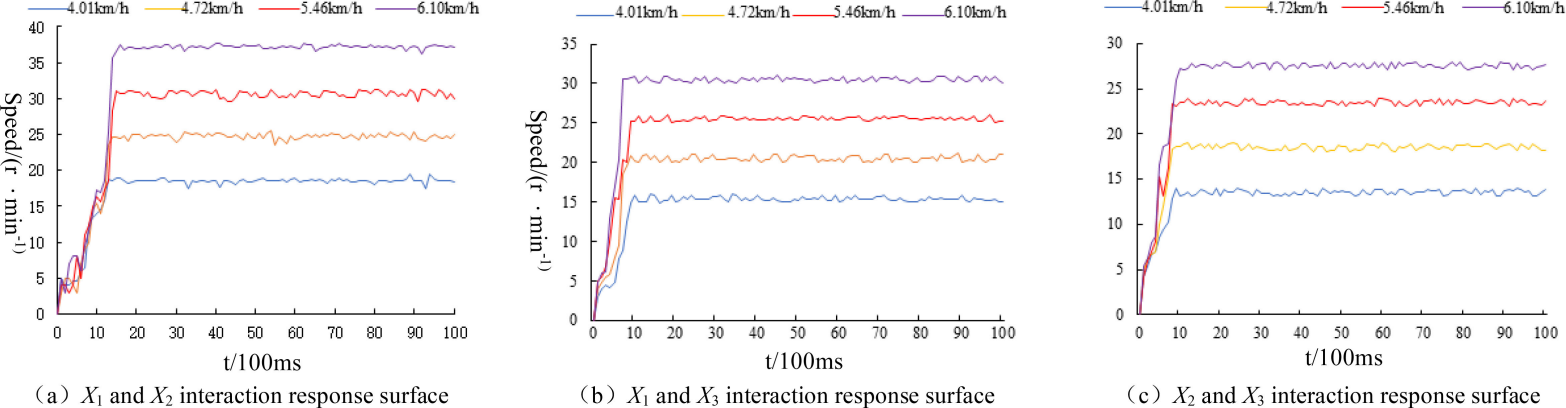
Speed response curves of seeding discharge motor. **(A)** *X*_1_ and *X*_2_ interaction response surface. **(B)** *X*_1_ and *X*_3_ interaction response surface. **(C)** *X*_2_ and *X*_3_ interaction response surface.

The mean velocity of the field-tested tractor in gears I~IV (fixed throttle) is approximately 4.01~6.10km·h^−1^. The results of the seed displacer speed response are illustrated in **Figure 16**. A slight fluctuation is observed when the seed displacer reaches steady state speed. However, the overall speed is stable, with no overshooting. A stable speed output can be achieved, and the overall regulation time is maintained near 1 second. The regulation response time is shorter.

The control test accuracy test results are shown in **Table 6**. The steady-state speed error of the seed expeller is 0.26, 0.27, and 0.31 rpm, respectively, when the planter operates at different speeds. The average control accuracy of the seed expeller speed control is 98.98%, 98.82%, and 98.77%, respectively. The transmission error is less than 0.40 r/min, indicating high control accuracy and good system stability. With regard to the seeder speed response, it was observed that as the Working speed of the planter increased, the starting power of the corresponding motor drive increased, as did the starting slope of the corresponding seeder speed. Furthermore, the average time required to reach the corresponding steady-state speed in the test set spacing of 15 cm, 18 cm, and 20 cm was found to be 1. The response time is not more than 1.5 s, with a total steady-state speed error that is smaller and a driving error with the speed furrow control system that is less than 0.40 r/min. The performance of the speed-following seeding control system is sufficient to meet the precision seeding operation of the planter.

**Table 6:** Seeder speed control performance test results.

Seeding spacing/cm	Working speed/(km·h^-1^)	Time/s	Inaccuracies/(r·min^-1^)	Correct rate/%
15	4.01	1.21	0.16	99.12
	4.72	1.45	0.31	98.76
	5.46	1.52	0.39	98.77
	6.10	1.37	0.17	99.26
18	4.01	1.10	0.23	98.94
	4.72	0.96	0.30	98.51
	5.46	0.87	0.23	98.54
	6.10	1.20	0.31	99.27
20	4.01	0.96	0.35	98.21
	4.72	0.85	0.22	99.23
	5.46	0.90	0.38	98.42
	6.10	1.10	0.29	99.22

Upon acceleration, the control system regulates the rotational speed of the seed dispenser shaft to maintain a consistent operational velocity following seed dispensing. The system utilizes different plant spacing as the initial setting value, demonstrating high precision in speed tracking, rapid response, and minimal steady-state error in rotational speed across varying plant spacings and operational speeds.

#### 5.3.2 Field trials on the quality of sowing operations

In accordance with the test methods and indexes delineated in GB/T 6973-2005 Test Methods for Single Grain (Precision) Seeding Machine, the conformity index, replayabiltity index, and undercurrent index are selected as evaluation indexes. Furthermore, the field production test evaluations of rotary plowing, crushing, and stubble stubbing of precision cotton seed drill are conducted in two modes: fixed-speed drive and speed-driven drive, respectively. The formulas for the conformity index of furrow spacing, the replayabiltity index, and the undercurrent index are as follows:


(13) $$\left\{ \begin{array}{l} A=\frac{n_{1}}{N^{'}}\times 100\\ D=\frac{n_{2}}{N^{'}}\times 100\\ M=\frac{n_{0}}{N^{'}}\times 100 \end{array}\right.$$

where *A* is the Conformity index, %; *D* is the replayability index, %; *M* is undercurrent index, %; *n*_1_ is the number of misses; *n*_2_ is the number of reseeds, one; *n*_0_ is the number of passes; *N’* is the theoretical number of seeds.

The plant spacing should be set at 15 cm, with the furrowing machine operated at the designated speed. The furrowing distance should be 50 m, with the furrowing process continuing without interruption. At each speed setting, three rows should be randomly selected. The quantity of seeds counted in each trial was 251. If the measured grain spacing exceeds the set distance, it should be adjusted accordingly. In the event of a missed sowing, the grain spacing is to be increased by a factor of five for a single missed sowing, by a factor of more than two and a half for two missed sowings, and so on. In the event that the measured grain spacing is less than 0.5 times the set grain spacing, it is necessary to proceed with reseeding. Conversely, if the set grain spacing is between 0.5 times the set grain spacing and 1.5 times the set grain spacing, the result is deemed to be satisfactory. Each experimental group was subjected to three replicates, and the resulting means were calculated. The results of the aforementioned tests are presented in **Table 7**.

**Table 7:** Results of field sowing operation quality test.

Working speed/(km·h^−1^)	Constant speed drive			RPM drive with speed		
	Undercurrent index/%	Multiple index/%	Conformity index/%	Undercurrent index//%	Multiple index/%	Conformity index/%
4.01	4.92	3.36	91.72	1.95	4.23	93.82
	3.56	5.45	90.99	0.84	3.64	95.52
	5.34	6.25	88.41	0.97	4.50	94.53
4.72	6.35	5.76	87.89	1.90	5.24	92.86
	5.24	6.74	88.02	1.65	3.67	94.68
	5.94	5.87	88.19	1.45	4.32	94.23
5.46	4.67	4.32	91.01	1.94	3.78	94.28
	4.86	2.89	92.25	2.24	4.52	93.24
	5.36	4.51	90.13	2.57	3.56	93.87
6.10	4.87	3.67	91.46	2.89	5.16	91.95
	6.34	4.75	88.91	3.01	4.75	92.24
	4.45	4.02	91.53	2.46	5.24	92.3

The speed-driven furrow control system was observed to meet the design requirements and perform better than the traditional fixed-speed drive when the target plant spacing was set at 15 cm and the machine operated at varying speeds. The qualified grain spacing index exceeded 90% at speeds between 4.01 and 6.10 km·h^−1^, while the leakage and reseeding indices remained below 5% and 6%, respectively. These results demonstrate that the speed-driven method is more effective than the fixed-speed drive in achieving the desired grain spacing. The results demonstrate that the speed drive control system meets the specified design requirements and is more effective than the traditional fixed-speed drive. Furthermore, the speed drive transmission mode meets the quality standards set forth by the agricultural industry for precision furrowing operations.

### 5.4 Seedling emergence effect

**Figure 17:**
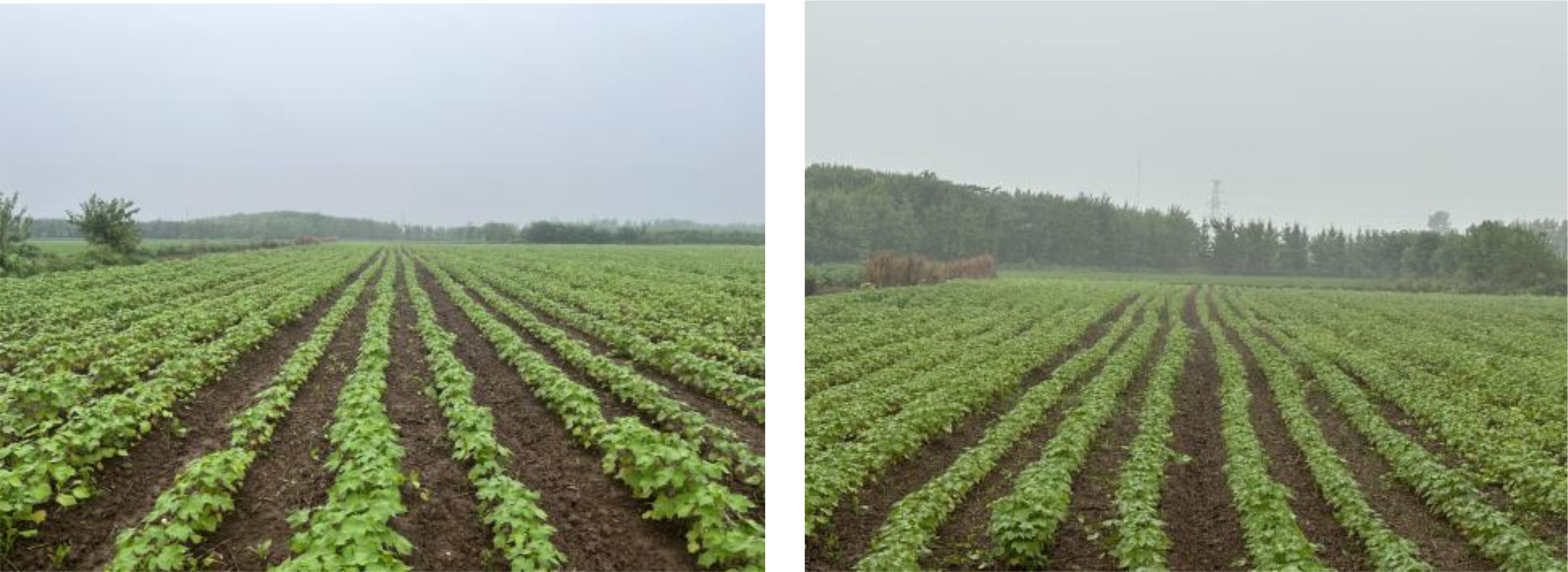
Field experiment.

To ascertain the efficacy of the entire machine in the field, a field sowing test was conducted on May 12, 2023. In the test, the theoretical grain spacing was set to 15 cm, the operation speed was set to 4.01-6.10 km·h^−1^, and the classical cotton seed Ezabu 30, which is widely cultivated in the Yangtze River basin, was used as the test area. The length of the test area was set to 50 m, and 20 m in the middle of each sown row(three rows in one pass) was taken as the data collection area. The test was conducted as a one-factor test, with each speed level repeated three times to obtain the mean value. Following a period of 30 days, the results are presented in **Figure 17**. In accordance with the national standard GB/T6973-2005, the quality evaluation of furrowing uniformity was conducted for the furrowing situation under different speeds. Upon examination of the speed change, it was determined that the sowing emergence spacing could be concentrated with in the set spacing within the range of qualified grain spacing(15 ± 3cm) emergence. This emergence exhibited consistency and uniformity. The results of seedling emergence and sowing performance are presented in **Table 8**. At vehicle speeds of 4.01-6.10 km·h^−1^, the system field sowing qualified at an average of 86.48%, with a minimum of 83.81%.

**Table 8:** Results of field sowing and seedling emergence experiments.

Working speed/(km·h^−1^)	Theoretical seeding spacing/cm	Sowing grain spacing qualification index/%	variation index/%
4.01	15	83.81	15.78
4.72	15	87.79	11.23
5.46	15	89.81	13.61
6.10	15	84.50	14.50

## 6 Conclusions

(1) The significance of the conformity index of furrowing depth in descending order is the operation speed, initial traction angle, and initial spring increment. The significance of the coefficient of variation of furrowing depth in descending order is the operation speed, initial spring increment, and initial traction angle. In the optimization test, the initial traction angle was set at -10°, the initial spring increment was 15mm, and the operational speed was 3 km·h^−1^. The qualified sowing depth index was 95.59%, and the coefficient of variation of sowing depth consistency was 7.38%. The results of the field test verification are as follows: the open furrow depth conformity index was 89.61%, and the open furrow depth consistency coefficient of variation was 10.54%. These results demonstrate that the depth of the open furrow meets the agronomic planting requirements.
(2) The cotton with speed furrowing control system is capable of meeting the agronomic requirements of precision furrowing in cotton. The speed of the system is 4.01 to 6.10 km·h^−1^, with a conformity index of more than 90% and a leakage of sowing and replanting index of less than 6%.
(3) The field test demonstrated that at speeds between 4.01 and 6.10 km·h^−1^, the average seedling emergence qualification (grain spacing) index was 86.48%, with a minimum of 83.81%. The spacing of cotton seedlings was observed to be stable and uniform. The effect of seedling emergence is favorable.

## Data Availability

The original contributions presented in the study are included in the article/**Supplementary Material**. Further inquiries can be directed to the corresponding author.
