# Supplementary material for: Design and testing of the key technology of the cotton direct seeding machine
Source: Front Plant Sci. 2025 Feb 20;16:1530725. doi: 10.3389/fpls.2025.1530725 (PMC11882871; doi:10.3389/fpls.2025.1530725)
Supplement: Supplementary file 1 [file DataSheet1.docx]

**Supplementary Material:**


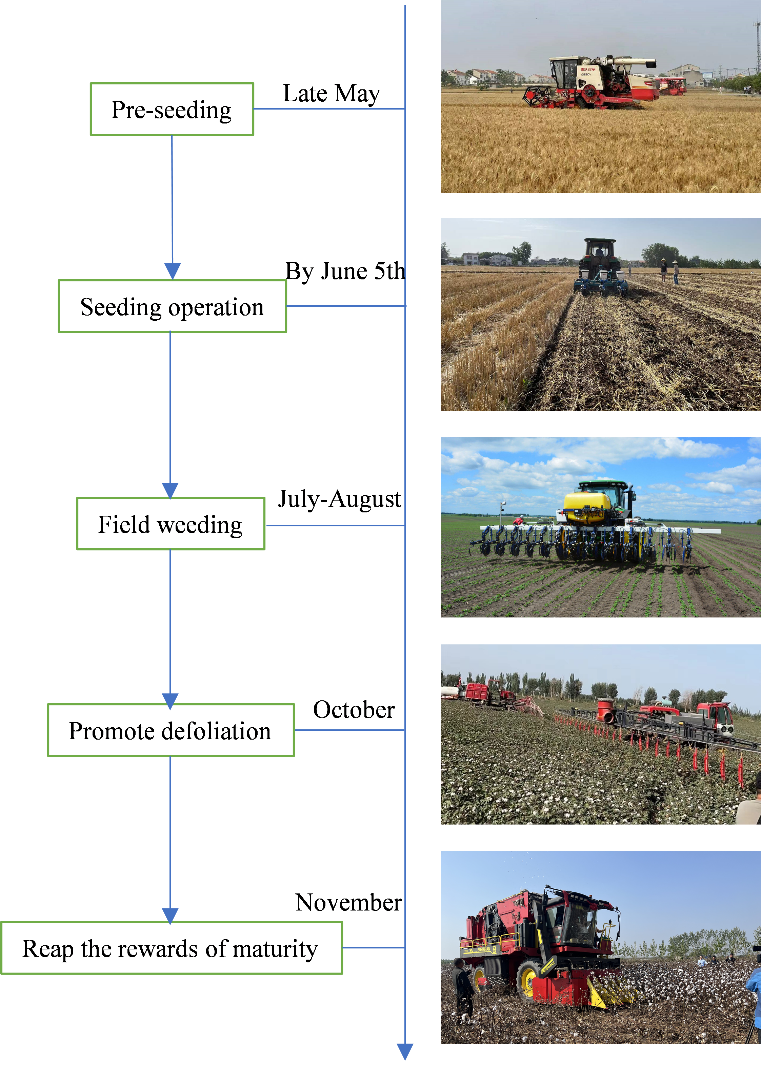


**Supplementary Figure 1.** Production technology requirements for post-wheat/oil direct seeding cotton planting pattern

**Supplementary Table 1.** Relationship between profiling angle and the effect of seeding depth

| α_0_/(°) | α_1_/(°) | α_2_/(°) | H/mm |
| --- | --- | --- | --- |
| 0 | 5 | 5 | 45 |
| 0 | 10 | 10 | 90 |
| 0 | 15 | 15 | 134.6 |
| 0 | 20 | 20 | 177.9 |

**Supplementary Table 2**. Results of verification experiment

| Order | X_1_ / ( °) | X_2_ / mm | X_3_ / ( km·h^-1^ ) | Y/% | V/% |
| --- | --- | --- | --- | --- | --- |
| 1 | 0 | 20 | 3.6 | 88.72 | 11.62 |
| 2 | 0 | 20 | 3.6 | 86.31 | 10.31 |
| 3 | 0 | 20 | 3.6 | 93. 8 | 9.69 |
